# New Luminescence Ages for the Galería Complex Archaeological Site: Resolving Chronological Uncertainties on the Acheulean Record of the Sierra de Atapuerca, Northern Spain

**DOI:** 10.1371/journal.pone.0110169

**Published:** 2014-10-22

**Authors:** Martina Demuro, Lee J. Arnold, Josep M. Parés, Alfredo Pérez-González, Ana I. Ortega, Juan L. Arsuaga, José M. Bermúdez de Castro, Eudald Carbonell

**Affiliations:** 1 Institute for Photonics and Advanced Sensing, School of Chemistry and Physics, The University of Adelaide, Adelaide, Australia; 2 Centro Nacional de Investigación sobre la Evolución Humana, Burgos, Spain; 3 The Environment Institute and School of Earth and Environmental Sciences, The University of Adelaide, Adelaide, Australia; 4 Centro Mixto Universidad Complutense-Instituto de Salud Carlos III de Evolución y Comportamiento Humanos, Madrid, Spain; 5 Departamento de Paleontología, Facultad de Ciencias Geológicas, Universidad Complutense de Madrid, Madrid, Spain; 6 Institut Català de Paleoecologia Humana i Evolució Social, Àrea de Prehistòria, Universitat Rovira i Virgili, Tarragona, Spain; University of Oxford, United Kingdom

## Abstract

The archaeological karstic infill site of Galería Complex, located within the Atapuerca system (Spain), has produced a large faunal and archaeological record (*Homo sp. aff. heidelbergensis* fossils and Mode II lithic artefacts) belonging to the Middle Pleistocene. Extended-range luminescence dating techniques, namely post-infrared infrared stimulated luminescence (pIR-IR) dating of K-feldspars and thermally transferred optically stimulated luminescence (TT-OSL) dating of individual quartz grains, were applied to fossil-bearing sediments at Galería. The luminescence dating results are in good agreement with published chronologies derived using alternative radiometric dating methods (i.e., ESR and U-series dating of bracketing speleothems and combined ESR/U-series dating of herbivore teeth), as well as biochronology and palaeoenvironmental reconstructions inferred from proxy records (e.g., pollen data). For the majority of samples dated, however, the new luminescence ages are significantly (∼50%) younger than previously published polymineral thermoluminescence (TL) chronologies, suggesting that the latter may have overestimated the true burial age of the Galería deposits. The luminescence ages obtained indicate that the top of the basal sterile sands (GIb) at Galería have an age of up to ∼370 thousand years (ka), while the lowermost sub-unit containing Mode II Acheulean lithics (base of unit GIIa) was deposited during MIS 9 (mean age = 313±14 ka; *n* = 4). The overlying units GIIb-GIV, which contain the richest archaeopalaeontological remains, were deposited during late MIS 8 or early MIS 7 (∼240 ka). Galería Complex may be correlative with other Middle Pleistocene sites from Atapuerca, such as Gran Dolina level TD10 and unit TE19 from Sima del Elefante, but the lowermost archaeological horizons are ∼100 ka younger than the hominin-bearing clay breccias at the Sima de los Huesos site. Our results suggest that both pIR-IR and single-grain TT-OSL dating are suitable for resolving Middle Pleistocene chronologies for the Sierra de Atapuerca karstic infill sequences.

## Introduction

**Figure 1:**
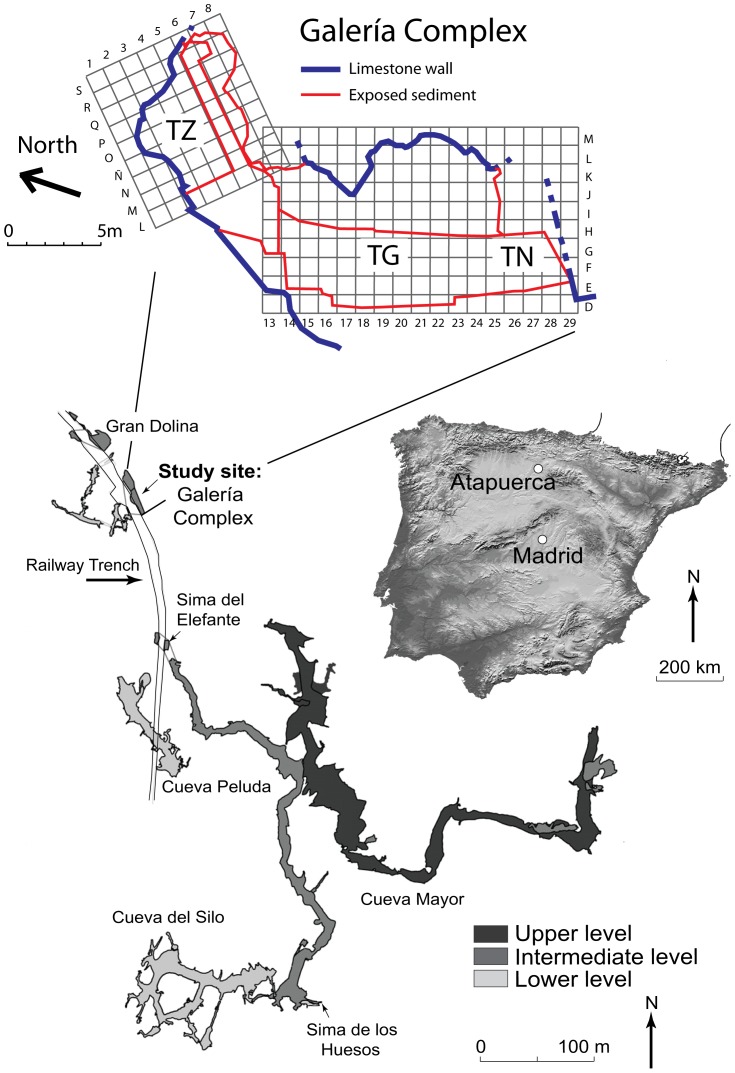
Geographical location of the Galería site within the abandoned railway trench at Atapuerca, Spain. The plan view of the Galería karstic complex shows the three conduits (Zarpazos: TZ, Galería: TG, and Tres Simas norte: TN) that have contributed to the infilling of the cavity.

The Sierra de Atapuerca archaeological complex, located in northern Spain, has been systematically excavated for the last 30 years and has provided a wealth of information for the Early to Middle Pleistocene palaeoanthropological record of Europe. Some of the most important findings at Atapuerca have included the unearthing of mandible ATE9-1 at Sima del Elefante, one of the oldest human remains of Western Europe (1.22±0.16 Ma) [1], the discovery of the largest accumulation of Middle Pleistocene hominin remains worldwide at Sima de los Huesos [2], which has also provided the oldest human mitochrondial DNA recovered thus far [3], and the excavation of >150 *Homo antecessor* remains from level TD6 at Gran Dolina [4]. These sites have also produced rich associated lithic artefacts and macro/micro mammal assemblages, which provide the necessary environmental and cultural context to reconstruct the nature of hominin occupation at Atapuerca [5], [6]. Key among the Atapuerca palaeoanthropological sites is the mid-late Middle Pleistocene site of Galería Complex, which represents one of the karstic infill exposures found along the abandoned railway trench and is located 50 m to the south of Gran Dolina and 100 m to the north of Sima del Elefante (**Figure 1**). This site contains various archaeo-palaeontological levels that have produced ∼12,000 large mammal remains, thousands of small vertebrates, ∼1800 lithic tools and two human skeletal fragments tentatively assigned to *Homo sp. aff. heidelbergensis* [6]–[9]. Examination of the large collection of Mode II lithics and abundant Acheulean large cutting tools (handaxes, cleavers), together with use-wear analysis and taphonomic investigations of preserved faunal remains, indicate that Galería was visited by humans for the exploitation of carcasses of large herbivores that had fallen down the vertical shaft at the southern end of the cavity [9], [10].

Numerical age constraint has been previously established at Galería using uranium-thorium (U-series) and electron spin resonance (ESR) dating of calcite/speleothems bracketing the sedimentary sequence [11]–[13], thermoluminescence (TL) and infrared stimulated luminescence (IRSL) dating of sedimentary silicates [14], and combined ESR/U-series dating of fossil teeth [15]. The existing luminescence chronologies, which are mostly based on TL dating, are systematically older than those obtained using the other methods. In particular, the TL/IRSL chronologies suggest that units GIIa-GIIIa (archaeological horizons TG7-TG10A) were deposited ∼500–440 ka, which corresponds to MIS 13/12 [14]. However, the combined ESR/U-series ages of mammal teeth from these units, as well ESR and U-series ages on intervening speleothems, indicate that the fossil accumulation occurred during MIS 9 through to MIS 8, and perhaps during early MIS 7 [15], [16]. The causes of the disagreement between the TL/IRSL ages and those ages obtained using alternative dating methods have not been clearly identified.

The importance of the Early-to-Middle Pleistocene archaeological records at Galería means there is a pressing need to resolve the chronological inconsistencies observed using different radiometric dating methods. This is also necessary because the polymineral IRSL/TL chronologies established at Galería and neighbouring sites (e.g., TD10; Gran Dolina) are often used as the basis for correlating the various archaeological and palaeontological records of Atapuerca [5], and are more broadly used to postulate hypotheses about the timing and nature of human occupation across Europe [17]–[20]. The episodic nature of sediment infilling and erosion in cave environments results in highly complex stratigraphic associations that are difficult to correlate temporally in the absence of direct numerical dating [21], as has been shown in recent studies [22], [23]. Improved site-specific chronological frameworks are therefore necessary for reliably reconstructing the karst system dynamics of the Atapuerca complex, as well as for constraining the spatio-temporal relationships of palaeoenvironmental proxies and biological and human remains trapped in caves, and for understanding the types of diagenetic processes that have affected these sedimentary sequences [24], [25]. For all these reasons, it is crucial to assess the accuracy, and expand the spatial coverage, of existing numerical chronologies established on the Atapuerca cave infills.

Luminescence dating of silicate minerals using visible light stimulation (optically stimulated luminescence, OSL) or infrared wavelengths (IRSL) offers advantages over many other chronological techniques because the material being dated is ubiquitously found in sedimentary environments [26]. In comparison to TL methods, OSL and IRSL techniques are also better suited for dating sedimentary deposits that have received relatively limited daylight exposure prior to burial because the signals employed can be reset by natural sunlight (either completely or to very low levels) in a matter of seconds to minutes [27]. The development of single-grain dating equipment, new measurement protocols, and improvements in statistical analyses of burial dose datasets [28], [29] have further enhanced the applicability of OSL and IRSL techniques by enabling incomplete signal resetting (partial bleaching) or post-depositional mixing to be more readily detected and appropriately treated in age calculations [30]–[33]. Quartz OSL dating has been successfully applied to sediments from a range of caves and rock shelters that contain associated archaeological records [34]–[36]. However, OSL is commonly only applicable to sediments that are <200 ka because the signal used for dating rapidly reaches saturation level (i.e., it does not continue to grow with additional radiation exposure) over moderate natural doses ranges.

The emergence of alternative luminescence dating techniques, such as thermally-transferred OSL (TT-OSL) dating of quartz and post-infrared IRSL (pIR-IR) dating of K-feldspars [37]–[40], offers new potential for circumventing the dose saturation restrictions of conventional OSL dating and for extending the upper age range of luminescence dating techniques over Middle Pleistocene timescales. Reliability assessments performed on these ‘extended-range’ luminescence techniques using known-age comparisons have produced encouraging results across a range of depositional contexts [33], [37], [39], [41]–[44]. There have also been several reliable TT-OSL and pIR-IR dating studies in potentially complex cave or rock shelter settings [35], [45]–[47]. However, a number of complications can potentially affect the accuracy of TT-OSL and pIR-IR ages, necessitating careful sample-specific suitability assessments in any dating study. In particular, there has been some debate over the long-term stability of the TT-OSL signal, with laboratory studies of electron-retention lifetimes suggesting that age underestimations may be a potential problem when dating very old (Early rather than Middle Pleistocene) samples [48]. There is also evidence to suggest that different types of pIR-IR measurement conditions may be required to obtain accurate ages for certain samples [42], [47], [49]. An additional complication with pIR-IR and TT-OSL dating is the slower rate at which these signals are optically bleached in comparison with conventional OSL signals [39], [50]–[52] (i.e., on the order of minutes-to-hours and weeks-to-months, respectively). These slower bleaching rates may increase the potential for insufficient signal resetting prior to deposition, particularly in the case of the very slowly bleaching TT-OSL signal. However, it has been recently shown that it is feasible to apply TT-OSL dating at the single-grain scale of analysis at the Atapuerca sites [33], [47], which permits detailed assessments of bleaching and post-depositional histories in the present context.

Here we report on results obtained using single-grain TT-OSL and multi-grain pIR-IR dating applied in tandem at the Middle Pleistocene site of Galería Complex. The aim of the paper is twofold. First, to generate new, systematic ages for the karstic infill sediment layers that have previously been dated in an attempt to resolve existing chronological inconsistencies. Second, to establish the first numerical age constraint on previously undated units at Galería and Covacha de los Zarpazos that underlie the archaeo-palaeontological levels. This extended-range luminescence study builds on similar comparative assessments undertaken at other Atapuerca sites [47]. We evaluate our findings with respect to these related studies and discuss the broader applicability of novel TT-OSL and pIR-IR techniques in similar cave settings.

### 1. Study Site - Galería

The Sierra de Atapuerca palaeoanthropological sites are located within a Cretaceous limestone karstic complex found 15 km east of Burgos, northern Spain (**Figure 1**). The sites are situated in the headwaters of the Duero basin and close to the Bureba corridor, which forms a connection with the Ebro basin to the east [53]. The Galería site (42° 21.08′N, 003° 31.19′W) is found within the intermediate zone (∼995 masl) of a multi-level karst system that formed during the Early Pleistocene by phreatic groundwater circulation and subsequent river downcutting of local base levels [54], [55] (**Figure 1**). Three separate conduits are thought to have contributed to the sedimentary infilling of the Galería Complex cavity, resulting in a complex sedimentary sequence characterised by strong lateral lithological variations. These conduits are associated with three sub-sections: Covacha de los Zarpazos (TZ) to the north; the main sub-horizontal gallery (Galería itself) in the centre (TG); and the northernmost infill segment (TN) of the neighbouring (and connected) Tres Simas cavity, which is located at the southern end of the Galería site [16], [56] (**Figure 1**).

### 2. Stratigraphy

**Figure 2:**
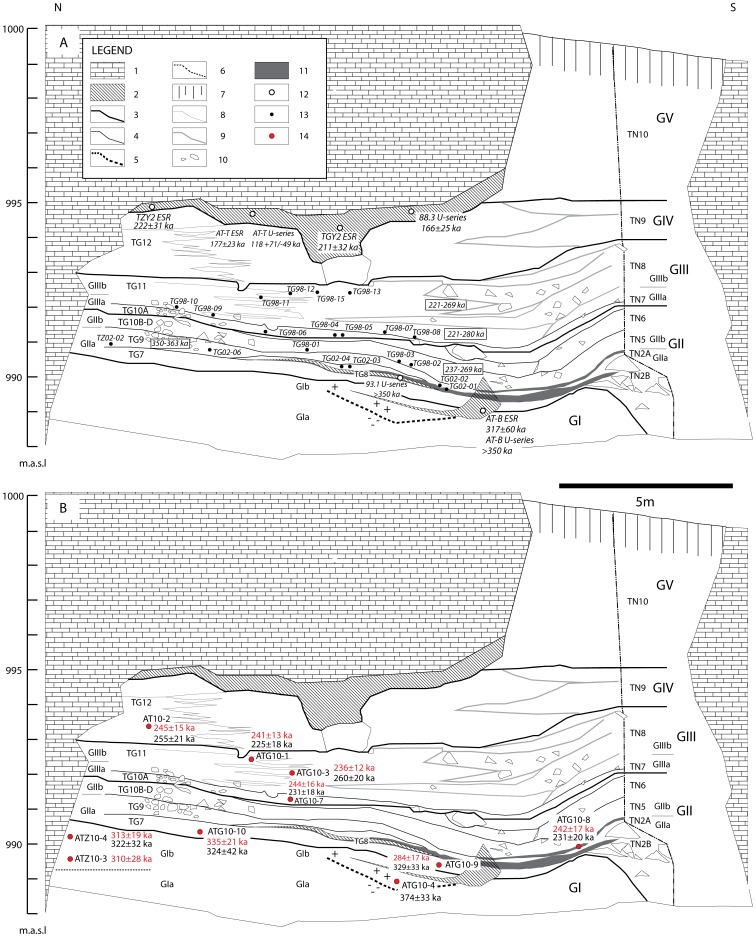
Stratigraphic sequence of the cave deposits at Galería Complex. (a) Ages/sample positions of teeth previously dated by combined ESR/U-series [15] and ages/sample positions of speleothem samples previously dated using U-series and/or ESR [11], [15], [104]. (b) Location of the luminescence dating samples and ages obtained in this study. Legend: (1) Cretaceous limestone; (2) Speleothem; (3) Main stratigraphic uncomformity – allostratigraphic unit (e.g., GII); (4) Lithologic/archaeo-palaeontological level (e.g., TG7); (5) Palaeomagnetic reversal; (6) Zarpazos – tentative GIa/GIb boundary; (7) Soil; (8) Clayey silt/small gravel boundary; (9) Gravels/breccia boundary; (10) Limestone clasts and boulders; (11) Organomineral layer (includes bat guano); (12) U-series and/or ESR speleothem samples; (13) ESR/U-series teeth samples; (14) Luminescence samples (this study).

The 6 to 13 m-thick stratigraphic sequence has been divided into five allostratigraphic units according to well-defined unconformities [12], [57] (**Figure 2**). From the base of the sequence upwards, the units are denoted as GI to GV (the uppermost unit contains an old edaphic horizon named TGVI). Unit GI is an internally-transported (endokarstic) sedimentary deposit, whereas GII to GV are allochthonous to the cave system. All of the palaeontological and archaeological finds have been collected from units GII and GIII; units GI and GIV-GV are sterile. A number of lithological levels have been identified in the north-central (TG) and southern (TN) parts of the cavity [58]. This stratigraphical framework was later used to delimit the main archaeo-palaeontological levels (TG7 to TG12 and TN2 to TN10) of Galería [10], [16], [59], though their exact relationships can be difficult to determine due to the spatial complexity of the stratigraphic sequence. The nomenclature adopted in this study for the allostratigraphic units and lithological/archaeo-palaeontological levels of Galería is based on published descriptions [15]. A summary of the sedimentological properties for each unit is given below and in **Table 1** following [12], [16], [57].

**Table 1:** Allostratigraphic units and lithological levels (same nomenclature used to describe archaeo-palaeontological levels) for Galería, together with the position of the luminescence dating samples analysed in this study.

Allostratigraphic unit	Thickness (m)	Description	Sub-units description	Lithological/Archaeo-palaeontological level		Luminescence dating Samples
				NORTH	SOUTH	
GV	5	Breccia composed of subangular gravels and blocks within a fine sandy silt and silty clay matrix [57]. Capped by a 1-1.5-m thick edaphic phase.		Sterile	Sterile	
GIV	1.5 to 2	Southern sector: Breccia deposits made up of angular limestone clasts supported by fine gravels and silt (<10%). Central sector: Dominated by well-sorted very fine subangular gravels (<3 cm) with <10% silt matrix. These deposits alternate with silts and clays to the north. Northern sector: Laminated sandy silts and clays with layers of small gravels or cemented mud balls.		TG12	TN9	AT10-2
GIII	1 to 2	The base displays an apparently massive fine sand and silt-clay layer up to 35 cm-thick that contains, at the extremities, gravels and up to 9–12 cm clasts (to the north). This deposit is overlain by three distinct infill facies: Southern sector: Breccia composed of angular limestone clasts supported by fine gravels and silty clay (<10%). Central sector: Succession of layers (up to15 cm-thick) composed of small (<3 cm) limestone gravels supported by a silty clay matrix, which are overlain by massive but occasionally laminated silty clays (up to 10 cm thick). Northern sector: Sandy silts and clays.	GIIIb: Presence of highly calcitic sands [62]	TG11	TN8	ATG10-1 (GSU 2), ATG10-3 (GSU 7)
			GIIIa: Encompasses the lower half of GIII and is characterised by the absence of calcitic sands.	TG11, TG10A	TN8, TN7	ATG10-7
GII	1 to 2.5	Yellowish sandy silts and clays with a 20–30 cm-thick dark grey organomineral horizon (containing bat guano). The organimineral layers (TN2A and TN2B) are intercalated with localised speleothems and clays. This horizon is overlain by breccias of rounded clasts (eroded and with double patinas) and laminated yellowish sands and reddish clays and silts. The breccias are overlain by clasts supported by gravels and clays	GIIb: Allochthonous sediments of reddish colour displaying significantly less post-depositional alternation of bones and sedimentary structures compared to GIIa.	TG10B-D	TN6, TN5	
		.	GIIa: Encompasses fine grained sediments and guano layers that display substantial post-depositional alteration and evidence of in situ lixivation (as in GIb), resulting in a lack of bone preservation in level TG7. Sub-unit contains microfacies composed of humate formation or peat, evidence of high biological activity and the presence of crandellite [62].	TG9, TG8, TG7	TN2A, TN2B	ATG10-8, ATG10-9, ATG10-10
GI	5	Internal facies (infill transported through the cave system rather than sourced from the immediate cave exterior). Laminated sands and silts with occasional planar and cross-stratification [57]. Deposition occurred prior to the roof collapse; as such this unit does not contain any fossils or archaeological remains.	GIb: Sands and silts with interstratified, sporadic speleothems and <4 cm-thick guano layers forming irregular laminar sequences [57]. High proportion of crandallite (3–30%) indicative of phosphate leaching from heavily altered bone and guano layers [57], [61].	Sterile	Sterile	ATG10-4, ATZ10-4, ATZ10-3
			GIa: Similar to GIb but separated by an unconformity. Palaeomagnetism indicates reversed polarity orientation.	Sterile	Sterile	

Based on [12], [15], [57]–[59], [62].

#### Unit GI

A 5-m thick facies dominated by light brown/yellow laminated fine sands and silts of fluvial origin that display occasional planar- and cross-stratification [57]. Deposition of this unit occurred prior to the roof collapse event(s) that subsequently enabled infilling of the cavity by material sourced from the immediate exterior of the cave. As such, this unit does not contain any fossils or archaeological remains and is thus considered to be autochthonous in the sense that it was transported through the endokarst system rather than being sourced directly from a nearby cave opening (note: this interpretation does not preclude an exterior origin for the sediments). A polarity reversal has been identified within this unit in the central sector [12] and has been attributed to the Matuyama-Brunhes (MB) boundary [60]. The upper section of GI, which has a normal polarity, is denoted GIb [16], while the lower, with reversed polarity, is termed GIa. The upper part of GI contains interstratified and sporadic speleothems and <4 cm-thick guano layers that form irregular laminar sequences [57]. The high proportion of crandallite (3–30%) within the clay fraction (<2 µm) of GIb and the lower section of GII (i.e., GIIa), indicates leaching of phosphates from the overlying/surrounding heavily altered bone and guano layers [57], [61].

#### Unit GII

This unit is separated from GI by an angular discontinuity and marks the beginning of the allochthonous sediment input. The infill has been sourced directly from the nearby cave exterior, as suggested by (i) the presence of collapsed limestone blocks towards the base of GIIa, corresponding with the opening of the TN shaft, (ii) a change in the colour of sediments towards reddish hues (5 YR 5/8), in agreement with the terra rossa deposits currently blanketing the surrounding landscape, and (iii) an increment in the amount of micro-morphological material originating from outside the cave [8], [16], [62]. The thickness of this unit ranges laterally from 1 m (to the north) to 2.5 m (to the south). Unit GII contains the first appearance of faunal remains and lithic artefacts at Galería and represents two phases of sediment infill. The first phase (GIIa) encompasses archaeo-palaeontological levels TG7 to TG9 and TN2B to TN2A, while the second phase (GIIb) contains levels TG10B-D and TN5 to TN6 [6], [16], [59]. The fragment of an adult human mandible containing M2 and M3 and assigned to *Homo sp. aff. heidelbergensis* [8] was uncovered from GII, although its exact stratigraphical position is unknown.

Sub-unit GIIa is distinguished by high levels of post-depositional alteration that are readily observable at the micro-morphological scale and that are absent in GIIb [62]. Within GIIa, high levels of crandellite point to lixivation of bat guano, which have resulted in the formation of weathering rings on limestones and significant bone destruction. The base of GIIa (level TG7) is laterally continuous and is composed of brown/yellow fine silts and clays devoid of clasts. The aformentioned alteration processes have prevented the preservation of much of the biological remains in this horizon, though numerous lithic tools attest to the presence of hominins during this time period [6], [10]. In the southern and central sectors of Galería, TG7 is overlain by two 5–30 cm-thick dark grey organomineral layers (containing bat guano) separated by red clays and silts (TN2A and TN2B) and a localised speleothem (TG8). In the northern sector, level TG7 is directly overlain by TG9, which is composed, from the base upwards, of brown-to-red fine silts and clays and an increasing abundance of small to medium size (<64 cm) limestone boulders. The second phase of GII (GIIb) was formed by an accumulation of clastic gravity deposits (i.e., debris fall) and contains abundant archaeo-palaeontological remains [16], [59]. Unit GIIb is composed mainly of boulders and gravels encased in red sandy silts and clays, or small nodules encased in a matrix of red silts and clays.

Prior to the opening of the TN shaft, which occurred sometime during the formation of GIIa, the environment inside the cave would have been characterised by semi-darkness and waterlogging, and was thus unsuitable for permanent hominin occupation [10]. However, the recovery of a lithic assemblage consisting of ∼200 pieces from GIIa (and ∼400 from GIIb; [63]) point to the presence and activities of hominins. It is inferred that hominins entered the cave for the attainment of animal parts (via a passageway), and subsequently transported and consumed these remains outside the cavity. The low number of refits and disproportionate abundance of shaped tools, medium-size flakes and percussion material throughout GII suggest that most of the knapping activities occurred outside the cave [6], further supporting the idea that the cave was visited for the collection of trapped animals.

A recent study of the lithic assemblage of unit GII [63] shows that both large cutting tools (e.g., handaxes and cleavers) and small- and medium-sized tools (e.g., denticulates and scrapers) are present in the assemblage (comprising ∼20% and ∼80%, respectively). Quartzite and Neogene chert are predominantly used in GIIa, but an increase in the exploitation of limestone, sandstone and Cretaceous chert is recorded in GIIb. Sub-unit GIIa is characterised by an almost exclusive use of cobbles as blanks for the production of large cutting tools, most of which are in the sharpening stages of configuration. Knapping techniques are mainly longitudinal and multipolar [63]. In contrast, sub-unit GIIb shows an increment in centripetal knapping techniques and the use of flakes as blanks, as well as a higher frequency of handaxes at the finishing stage of configuration. These trends indicate the use of longer operative chains to produce more extensively elaborated tools when compared to GIIa [63].

#### Unit GIII

This unit is separated from GII by a gentle unconformity and was deposited via gravitational mass movement and slopewash processes. The preserved sedimentary features indicate that water transportation and alluvial deposition were more dominant with this unit. The base of GIII is composed of a massive fine sand and silt-clay layer that displays a higher frequency of gravels and blocks towards the southern and northern margins. This is overlain by a deposit characterised by marked lateral variations owing to influences of different sedimentary inputs from the northern (TZ), central (TG) and southern (TN) sector conduits (see **Table 1** for details). GIII has been divided into GIIIa (lower) and GIIIb (upper) sub-units based on the presence of calcitic sands in the upper half of the deposit [62]. GIIIa corresponds to archaeo-palaeontological levels TG10A, the base of TG11 and TN7. The upper sub-unit of GIII (GIIIb) is composed of archaeo-palaeontological levels TN8 and upper TG11. Twelve occupation floors have been identified within unit GIII, indicating repeated exploitation of the cave for the acquisition of pitfall carcasses. These floors contain abundant paleontological and archaeological remains and collectively form archaeo-palaeontological level TG11 (GSU1-12). A human neurocranial fragment was uncovered during 1995 from the base of GIII [7]. In terms of the lithic assemblage [6], most of the Acheulean implements from GIII were derived from large flakes, and the raw materials were predominantly limestone, sandstone and Cretaceous chert. As with GII, small- and medium-sized shaped tools (denticulates, scrapers, etc) make up a substantial part of the identifiable lithics recovered from GIII (∼80–85%; see Tables 10 and 12 of [6]).

#### Units GIV to GV

These remaining units do not contain any archaeological or palaeontological remains. Unit GIV sits unconformably on GIII and is of similar lithology. This unit corresponds to lithological levels TG12 and TN9. Unit GV represents the final infilling of the vertical conduit to the south and comprises 6 or 7 clastic mass movement episodes. The sediments of GV are primarily subangular gravels separated by fine sandy silt and silty clay layers [57]. The top of unit GV contains one of the oldest edaphic phases preserved in the Atapuerca hills.

### 3. Previous chronological work

#### 3.1. Radiometric dating and palaeomagnetism

The ages obtained previously at Galería are summarised in **Figure 2a** and **Table 2**. The entire archaeo-palaeontological sequence is bracketed by the MB geomagnetic reversal in the lowermost level (GI) [60], and an U-series and ESR dated speleothem crust (stalagmitic floor) overlying GIV. This stalagmitic floor has produced the following radiometric ages: 118+71/−49 (U-series, [11]), 166±25 ka (U-series, cited in [15]), 177±23 ka (ESR, [11]) and 211±32 ka (ESR, [13]). Another stalagmite attached to the Zarpazos cave ceiling, which has been correlated with the capping crust of unit GIV, has also produced an age of 222±31 ka (ESR, [13]). Three additional speleothems have been dated from the horizons associated with units GI to GIII at Galería. A speleothem from a section now absent at the neighbouring Tres Simas site (labelled TS [15]), which has been stratigraphically correlated with the boundary between GIIIa and GIIIb, has been dated to 256±33 ka (ESR, [13]). Another speleothem located in unit GIIa (TG8) was found to be in isotopic equilibrium and yielded a minimum U-series age of >350 ka (Bischoff unpublished data; reported in [15]). Finally, in the upper section of GI, a third speleothem has been dated to >350 ka and 318±60 ka by U-series and ESR, respectively [11]. Relatively few details have been provided about the ESR and U-series speleothem ages obtained on units GI to GIV at Galería. Consequently, it remains difficult to assess any potential variations in methodological reliability from the published literature.

**Table 2:** Final luminescence ages obtained for the Galería samples in this study.

Allostratigraphic unit		Lithological/Archaeo-palaeontological level	Speleothems			Polymineral fine grain-Additive dose *				ESR/U-series on teeth		This study		
			Sample	Age (ka)		Sample	Age (ka)			Sample	Age (ka)	Sample	Age (ka)	
				ESR	U-series		IRSL	TL	IRSL and TL				SG TT-OSL	pIR-IR_225_
Stalagmite			AT-T	177±23 **	118+71/−49 **									
			TGY2	211±32^#^										
			TZY2	222±31^#^										
			88.3		166±25 **									
GIV		TG12				98–19	1340±170							
		TG12				97–14	185±26	510±100				AT10-2	255±21	245±15
GIII	GIIIb	TG11 (GSU2)				98–17			269±27			ATG10-1	225±18	241±13
		TG11 (GSU3)								TG9815	221 +15/−12			
		TG11 (GSU3)								TG9813	234 +20/−19			
		TG11 (GSU3)								TG9812	238 ±19			
		TG11 (GSU4)								TG9811	269 +51/−44			
		TG11 (GSU7)				97–24		224±42				ATG10-3	260±20	236±12
Speleothem	TS-S^a^		TSY12	256±33^#^										
	GIIIa	TG11 (GSU11A)				98–15		480±48		TG9810	280 +30/−27			
		TG10 (GSU12)								TG9807	231 +25/−24			
		TG10 (GSU12)								TG9809	233 +30/−28			
		TG10 (GSU12)								TG9808	244 +49/−26^b^			
		TG10A				97–19		439±66		TG9806	256 +26/−25^b^	ATG10-7	231±18	244±16
		TG10A								TG9805	227 +34/−34			
		TG10A								TG9804	239 +26/−24			
GII	GIIb	TG10D								TG9803	237 +26/−24			
		TG10D								TG9802	262 +35/−34			
		TG10D								TG9801	269 +26/−24			
	GIIa	TN2A										ATG10-8	231±20	242±17
TZ		(TG9)								TZ0202	363 +44/−42			
	GIIa	TG9				98–12		422±55		TG0206	350 +47/−46			
		TN2B								TG0201	217 +31/−28			
		TG8								TG0203	244 +56/−47			
		TG8								TG0202	274 +28/−26^b^			
		TG8								TG0204	211 +18/−16			
		TG8	93.1		>350 **									
		TG7				97–6		503±95				ATG10-9	329±33	284±17
		TG7										ATG10-10	324±42	335±21
TZ												ATZ10-4	322±32	313±19
												ATZ10-3		310±28
Speleothem			AT-B	318±60 **	>350 **									
GI	GIb					97–2		3000±1800				ATG10-4	374±33	
	GIa													

Also shown are the ages obtained for the infill sequence using TL and IRSL dating of (*) polymineral fine grains [14], (**) U-series and ESR dating of speleothems [11], [15], (#) ESR dating of speleothems [104], and combined ESR/U-series dating of herbivore teeth [15].

a TS-S refers to the location of the speleothem at Tres Simas Sur (see [15] for details).

b Samples having potentially complex uranium leaching and uptake histories according to [15]. Errors on ages are reported at 1σ.

Multiple aliquot, polymineral fine-grained luminescence dating results for the main sedimentary sequence at Galería have been reported [14]. The TL and IRSL ages obtained in this study showed a wide range of values, some of which were stratigraphically reversed and/or in disagreement with associated palaeomagnetic, U-series and ESR chronologies (see Table 2 of [14]). A number of these results (5 of the 15 ages) were excluded from the final age summary as they were deemed to be affected by signal saturation (in the case of IRSL signals) or partial bleaching (in the case of slower-bleaching TL signals). The resultant luminescence chronological framework suggested that units GIV and GIIIb were deposited ∼190–260 ka, which is in broad agreement with the aforementioned intervening speleothem ages for units GIV and GIIIb. The TL samples that were considered to be reliable for the lower units GIIIa and GII yielded ages of between ∼420 ka and ∼500 ka (**Table 2**).

Most recently, 20 ESR/U-series ages have been presented for large herbivore teeth from various levels of units GII and GIII [15]. Bayesian modelling was applied to the resulting ages to produce a chronological framework for the stratigraphical units at Galería that contain archaeo-palaeontological remains. The combined age range calculated for GIIIb, GIIIa and GIIb were 269–221 ka (*n* = 4), 280–221 ka (*n* = 7) and 269–237 ka (*n* = 3), respectively. For unit GIIa, the modelled ages increase significantly to 363–350 ka (*n* = 2), indicating a temporal hiatus between GIIa and GIIb. However, four additional teeth ages of between ∼270 and ∼211 ka were obtained for unit GIIa (TN2B and TG8) in close contact with the guano layer. These younger ages were excluded from the Bayesian age modelling as they were deemed to exhibit complex uranium leaching and uptake histories. The final ESR/U-series chronologies (minus the few young ages) indicate that units GIII and GIIb were deposited between 220–280 ka, while unit GIIa was deposited substantially earlier at 350–363 ka [15].

#### 3.2. Biochronology

The micromammal assemblages from GII and GIII are ascribed to Atapuerca Faunal Unit 6 (FU6) [64]. This faunal unit is represented by *Terricola atapuerquensis*, *Iberomys brecciencis*, *Microtus arvalis*, *Microtus argestis jansoni* and *Pliomys lenki*. The Galería assemblage can therefore be confidently placed within the I. brecciensis Biozone for the Spanish Pleistocene [64], which is defined by the presence of *I. brecciencis* and *T. atapuerquensis*. This micromammal biozone covers a relatively broad age range from 780 to 100 ka. Biometric analysis of *I. brecciencis* molars indicates the presence of a discontinuity between the lower levels of GII with respect to upper GII and GIII [65]. The large mammal assemblage is composed of species typical of the European Middle Pleistocene, such as the carnivores *Panthera leo*, *Vulpes vulpes*, *Canis lupus* and *Cuon alpinus europaeus*, and the hervibores *Dama dama clactoniana*, *Cervus elaphus*, *Stephanorhinus hemitoechus* and *Equus ferus* [5]. The composition and relative abundance of large mammal species are similar throughout the various levels of the Galería sequence (GII-GIII), suggesting that infilling of the cavity and accumulation of the fossils occurred within a relatively short lapse of time (i.e., within the same glacial-interglacial cycle) [16], [66].

## Materials and Methods

### 1. Luminescence dating sample details

**Figure 3:**
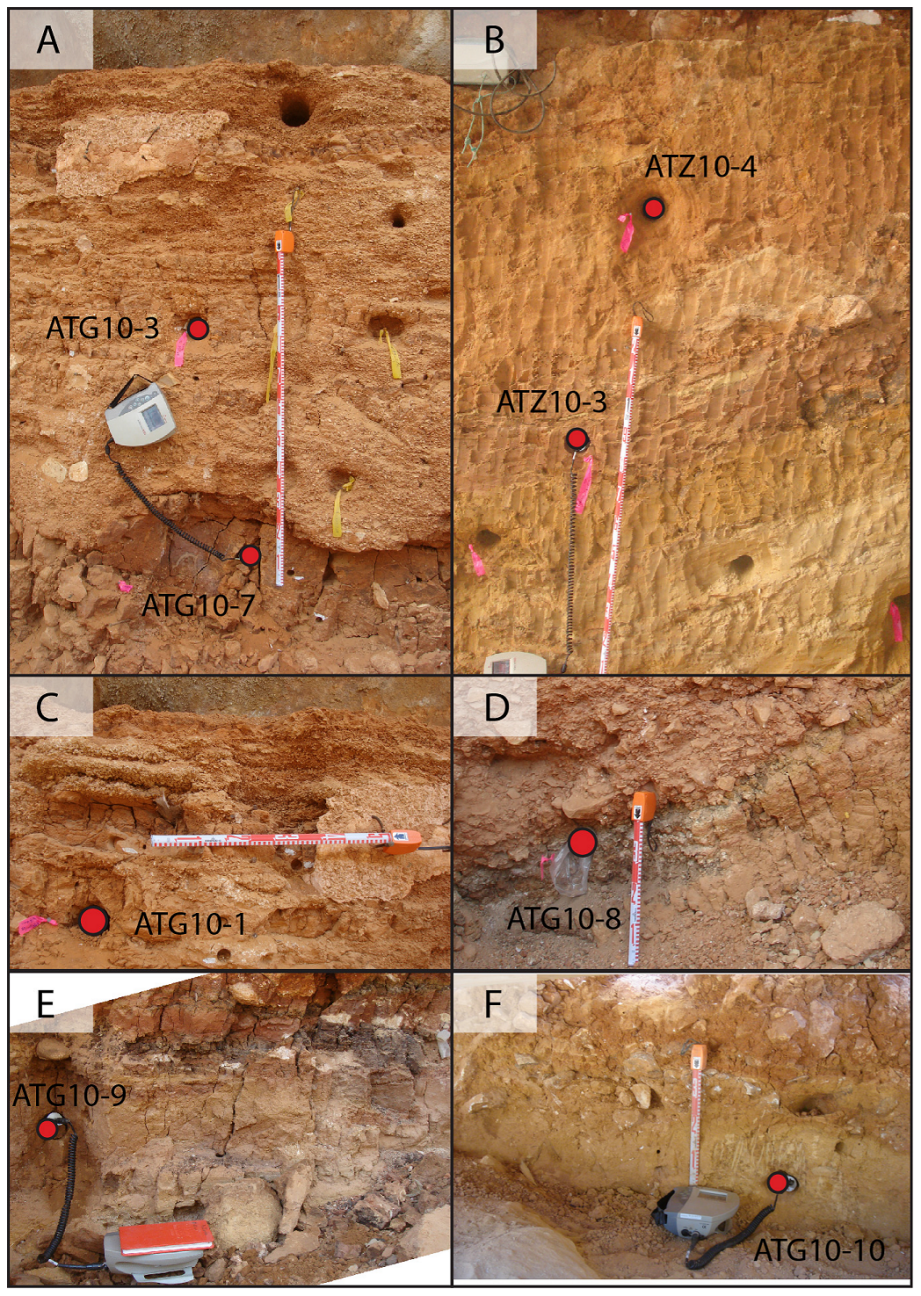
Photos showing the location of the luminescence dating samples and the types of deposits investigated at Galería. (a) and (c) show the sampling positions in unit GIII; (b) shows the stratigraphic sequence sampled at Covacha Zarpazos (northern wall); (d) sampling of the upper guano in unit GIIa (level TN2A); (e) and (f) shows sampling of the lower part of unit GIIa (level TG7).

Access and permission to collect samples in Atapuerca was granted by the Junta de Castilla y León, Spain. Ten samples were collected from units GI to GIV, as shown in **Figure 2b** and **Figure 3** and **Table 1**. Units, or lenses within units, dominated by homogenous sandy silts and clays were targeted for sampling. We specifically avoided locations that were affected by cementation, carbonate/calcite growth and (where possible) those with non-homogenous compositions/grain sizes to minimise the potential effects of beta-dose heterogeneity (see Table S1 in File S1 for lithological descriptions of each sample). One sample was collected from unit GIV (AT10-2) and two were collected from GIIIb (ATG10-1 and ATG10-3), within fine silts and clays towards the northern (Zarpazos) side of the profile (**Figure 3a, c**). During sampling of sub-unit GIIIb, care was taken to avoid layers that were predominantly composed of small limestone gravels and cemented calcitic sands. Specifically, samples ATG10-1 and ATG10-3 were collected from two thin (<10 cm) layers of sandy silts and clays corresponding to GSU 2 and GSU 7, respectively, which intercalate gravelly layers towards the central northern sectors. The base of GIII (GIIIa) was sampled within a massive horizon of homogenous fine silt and clay preserved in the centre of the profile (ATG10-7; **Figure 3a**). Three samples were collected from GIIa; one from the upper guano layer in sub-unit TN2A, which is underlain directly by red clays and fine silts (ATG10-8; **Figure 3d**), and another two samples from the base of GIIa within TG7, which is composed of brown/yellow sandy silt and clay (samples ATG10-9 and ATG10-10; **Figure 3e-f**). Two samples were collected from a level at Zarpazos that lies directly below the archaeo-palaeontological levels at Galería (i.e., below GII) and is stratigraphically correlated as lying at the top of unit GI (samples ATZ10-3 and ATZ10-4; **Figure 3b**). The upper part of unit GI (GIb) was sampled in the central sector of Galería (sample ATG10-4) within a horizon bracketed by the palaeomagnetic reversal and an overlaying speleothem that has been dated to >350 ka and 318±60 ka by U-series and ESR, respectively.

### 2. Luminescence dating methods

Luminescence dating was carried out at the CENIEH Luminescence Dating Laboratory, Burgos, Spain. Pure coarse-grained quartz (90–125 µm) and K-feldspar fractions (90–125 µm) were extracted using standard preparation procedures [26]. The outer (∼10 µm) alpha-irradiated rinds of the quartz extracts were etched with a treatment of 48% hydrofluoric (HF) acid for 45 minutes. For K-feldspars, etching was done using 10% HF for 10 minutes. The etched fractions were treated with 30% HCl for 45 minutes to eliminate any acid-soluble fluoride precipitates, and sieved again (using a 63 µm sieve) to remove any disaggregated or partially etched grains.

The external dose rates were calculated using a combination of *in situ* gamma-ray spectrometry and low-level beta counting (**Table 3**). The beta dose rate measurements were made on dried and homogenised material collected directly from the luminescence sampling positions. Beta dose rate measurements were made using a Risø GM-25-5 beta counter. Gamma dose rates were determined from *in situ* gamma-ray spectrometry measurements to account for any spatial heterogeneity in the surrounding gamma radiation field. Radionuclide concentrations of U, Th and K were derived from the gamma-ray spectra using the approach described in [67] and published conversion factors [68]. Cosmic-ray dose rates were calculated according to theoretical calculations [69] after taking into consideration site altitude, geomagnetic latitude, and density, thickness and geometry of sediment and bedrock overburden. The state of secular equilibrium in the ^238^U and ^232^Th decay series was assessed via high resolution gamma-ray spectrometry (HRGS) measurements (Table S2 in File S1). The daughter-parent isotopic ratios for ^238^U, ^226^Ra, ^210^Pb, ^228^Ra and ^228^Th indicate that the ^238^U and ^232^Th chains of the measured samples are in present-day secular equilibrium (ratios are consistent with unity at either 1σ or 2σ).

**Table 3:** Environmental dose rate values for the quartz and K-feldspar fractions measured in this study.

				Environmental dose rate (Gy/ka)^b^							
Sample	Sample depth (m)	Grain fraction (µm)	Water content^a^	Gamma dose rate^c^	Beta dose rate^d^	Cosmic dose rate^e^	Internal dose rate for	Internal dose rate for	Internal dose rate for	Total dose rate for Qz	Total dose rate for K-feldspars
							Qz	K-feldspars	K-feldspars	(Gy/ka)^h^	(Gy/ka)^h^
							(U+Th)^f^	(U+Th)^f^	(K+Rb)^g^		
AT10-2	7.01	90–125	16.0	0.69±0.03	1.53±0.07	0.07±0.01	0.03±0.01	0.06±0.03	0.43±0.03	2.32±0.12	2.78±0.13
ATG10-1	7.75	90–125	19.2	0.65±0.02	1.53±0.08	0.06±0.01	0.03±0.01	0.06±0.03	0.43±0.03	2.27±0.13	2.73±0.14
ATG10-3	8.15	90–125	16.7	0.59±0.02	1.52±0.07	0.06±0.01	0.03±0.01	0.06±0.03	0.43±0.03	2.20±0.12	2.66±0.12
ATG10-7	9.00	90–125	22.1	0.95±0.03	1.57±0.08	0.06±0.01	0.03±0.01	0.06±0.03	0.43±0.03	2.60±0.16	3.06±0.16
ATG10-8	10.10	90–125	30.8	0.95±0.03	1.34±0.02	0.05±0.01	0.03±0.01	0.06±0.03	0.43±0.03	2.37±0.17	2.83±0.18
ATG10-9	10.80	90–125	22.0	1.11±0.04	1.63±0.09	0.05±0.01	0.03±0.01	0.06±0.03	0.43±0.03	2.81±0.17	3.27±0.18
ATG10-10	10.60	90–125	22.0	0.96±0.04	1.47±0.08	0.05±0.01	0.03±0.01	0.06±0.03	0.43±0.03	2.51±0.15	2.97±0.16
ATZ10-4	10.00	90–125	24.9	1.00±0.03	1.83±0.10	0.05±0.01	0.03±0.01	0.06±0.03	0.43±0.03	2.91±0.19	3.37±0.19
ATZ10-3^i^	10.40	90–125	26.0	1.06±0.03	2.05±0.12	0.05±0.01	-	0.06±0.03	0.43±0.03	-	3.65±0.22
ATG10-4^i^	11.10	90–125	20.4	1.00±0.03	1.48±0.08	0.05±0.01	0.03±0.01	-	-	2.56±0.25	-

a Long-term water content, expressed as % of dry mass of sample and assigned a relative uncertainty of ±20%. Long-term water contents are calculated as 60% of saturated values following assessments made in deeper parts of the endokarst system [47].

b Radionuclide concentrations and specific activities have been converted to dose rates using the conversion factors given in [68], [105], making allowance for beta-dose attenuation [106], [107].

c Gamma dose rates were calculated from *in situ* measurements made at each sample position with a NaI:Tl or LaBr_3_:Ce detector using the ‘energy windows’ method [67].

d Beta dose rates were calculated using a Risø GM-25-5 low-level beta counter [108], after making allowance for beta dose attenuation due to grain-size effects and HF etching [107].

e Cosmic-ray dose rates were calculated following published procedures [69] and assigned a relative uncertainty of ±10%.

f Assumed internal (alpha plus beta) dose rate for the quartz fractions are based on published ^238^U and ^232^Th measurements for etched quartz grains from a range of locations [109]–[112] and an alpha efficiency factor (a-value) of 0.04±0.01 [113], [114]. For K-feldspar grains, the internal alpha and beta dose rate contributions from ^238^U and ^232^Th were calculated using assumed concentrations of 0.15±0.03 ppm and 0.35±0.07 ppm, respectively, based on modal values [112] and similar published values [115]–[117]. An a-value of 0.09±0.03 was used to estimate the internal alpha dose rate contributions from these ^238^U and ^232^Th concentrations based on published estimates obtained for a wide range of K-feldspar samples [14], [113], [118]–[121].

g Internal dose rate of feldspar grains arising from ^40^K and ^87^Rb concentrations were calculated using assumed values of 12.5±0.5% [122] and 400±100 ppm [123], respectively.

h Mean ± total uncertainty (68% confidence interval), calculated as the quadratic sum of the random and systematic uncertainties.

i TT-OSL measurements have not been made on sample ATZ10-3 and pIR-IR measurements have not been made on sample ATG10-4. Hence, only the relevant quartz or K-feldspar dose rates are shown for these two samples.

Luminescence signals were measured using a Risø TL-DA-20 reader [70]. Quartz and K-feldspar stimulations were performed with blue LED units (470±20 nm, maximum power of 84 mW cm^−2^) and an array of infrared (IR) LEDs (875 nm, maximum power of 151 mW cm^−2^), respectively. Single-grain stimulations were performed using a 10 mW Nd:YVO4 single-grain laser attachment emitting at 532 nm (maximum power of ∼50 W cm^−2^). Luminescence signals were detected using an EMI 9235QA photomultiplier tube. The ultraviolet quartz emissions were measured through a 7.5 mm-thick Hoya U-340 filter. Blue emissions from K-feldspar were measured through a filter combination pack composed of a 3 mm-thick Schott BG39 and a 4 mm-thick Corning 5–79. Samples were irradiated using a calibrated ^90^Sr/^90^Y beta source mounted on the reader. For single-grain measurements, the beta source had been calibrated to administer known doses to each grain-hole position across the disc plane.

TT-OSL and pIR-IR equivalent dose (D_e_) values were measured using the single-aliquot regenerative-dose (SAR) protocols shown in **Table 4**. TT-OSL measurements were made on individual quartz grains rather than on multi-grain aliquots, following the successful application of this approach elsewhere at the Atapuerca sites [33], [47]. Quartz D_e_ estimates were determined using a published TT-OSL SAR protocol [71], which has been modified to enable measurements at the single-grain scale of analysis (**Table 4a**). K-feldspar D_e_ values were measured using a multi-grain aliquot pIR-IR SAR protocol [72]. This D_e_ measurement protocol (herein denoted pIR-IR_225_) involves pIR-IR stimulation temperatures of 225°C and regenerative-/test-dose preheat temperatures of 250°C for 60 s (**Table 4b**). Further details of the D_e_ measurement procedures, including dose-recovery suitability tests and the rejection criteria used to eliminate unreliable grains and aliquots, are provided in the Supporting Information (see results in Table S4 and Table S5).

**Table 4:** SAR protocols used in this study to obtain ages from quartz (protocol A) and K-feldspars (protocol B).

A			B		
Step	Single-grain TT-OSL SAR protocol		Step	SAR pIR-IR multi-grain aliquots	
1^a^	Give dose		1^a^	Give dose	
2	Preheat to 260°C for 10 s		2	Preheat to 250°C for 60 s	
3	Stimulate with green laser at 125°C for 2 s (90% power)		3	Stimulate with infrared diodes 50°C for 200 s (90% power)	
4	Preheat to 260°C for 10 s		4^b^	Stimulate with infrared diodes 225°C for 200 s (90% power)	pIR-IR
					L_n_ or L_x_
5	Stimulate with green laser at 125°C for 2 s (90% power)	TT-OSL	5	Give test dose (100 Gy)	
		L_n_ or L_x_			
6	Stimulate with blue LEDs at 280°C for 400 s		6	Preheat to 250°C for 60 s	
7	Give test dose (200–300 Gy)		7	Stimulate with infrared diodes 50°C for 200 s (90% power)	
8	Preheat to 260°C for 10 s		8^b^	Stimulate with infrared diodes 225°C for 200 s (90% power)	pIR-IR
					T_n_ or T_x_
9	Stimulate with green laser at 125°C for 2 s (90% power)		9^c^	Return to 1	
10	Preheat to 260°C for 10 s				
11	Stimulate with green laser at 125°C for 2 s (90% power)	TT-OSL			
		T_n_ or T_x_			
12	Stimulate with blue LEDs at 290°C for 400 s				
13	Return to 1				

L_n_ and L_x_ refer to the natural and regenerative-dose signal measurements, respectively. T_n_ and T_x_ refer to the test dose signals measured after the L_n_ and L_x_ signals, respectively. Each SAR measurement cycle was repeated for the natural-dose, three to four different sized regenerative doses, a 0 Gy regenerative-dose (to measure OSL signal recuperation) and a replicate of the first regenerative-dose cycle (to assess the suitability of the test-dose sensitivity correction). In the case of the single-grain TT-OSL SAR procedure, the OSL IR depletion ratio [124] was measured separately and used to check for the presence of feldspar contaminants.

a Step omitted when measuring the natural signal (L_n_).

b Aliquots were held at pIR-IR stimulation temperatures for 10 s prior to switching on the IR diodes to avoid any potential isothermal TL contamination of the pIR-IR signal.

c A high temperature IRSL stimulation was not included at the end of each pIR-IR SAR measurement cycle because these samples did not display significant signal recuperation (the 0 Gy regenerative dose pIR-IR signal was consistently <3% of the sensitivity-corrected natural pIR-IR signal for all measured aliquots).

## Results

### 1. TT-OSL and pIR-IR signal characteristics

**Figure 4:**
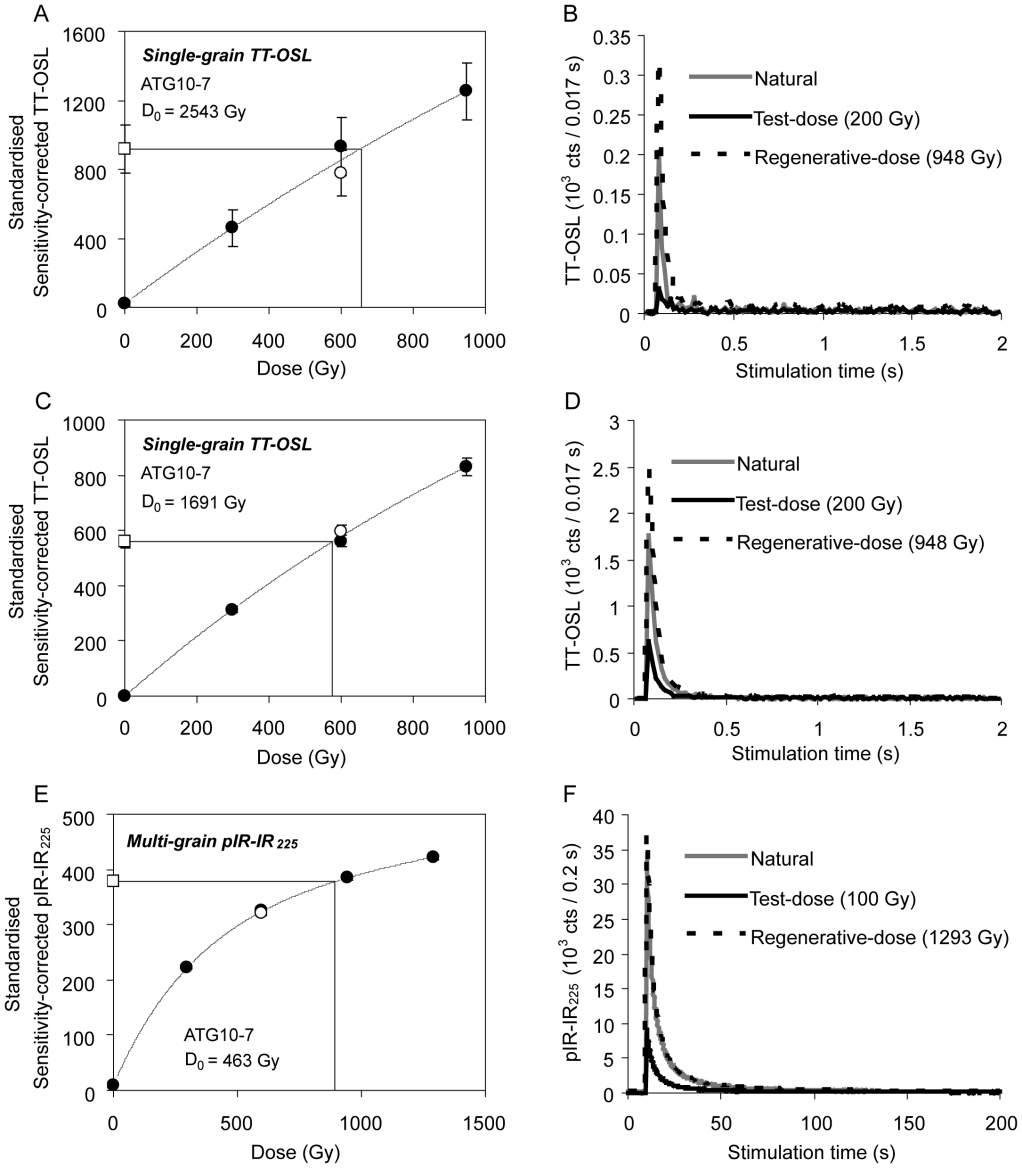
Selected examples of dose-response curves and signal decay curves. (a-d) TT-OSL single-grain measurements and (e-f) pIR-IR_225_ measurements of the Galería samples.

Representative TT-OSL decay curves and sensitivity-corrected dose-response curves are shown in **Figure 4a-d** for two accepted grains from unit GIIIa (sample ATG10-7). These two grains were measured from the same single-grain disc but display very different luminescence sensitivities; one has a relatively bright TT-OSL signal (T_n_ intensity >1500 cts/0.02 s) (**Figure 4d**), while the other displays a dimmer T_n_ luminescence intensity of ∼200 cts/0.02 s (**Figure 4b**). The latter is representative of the majority of grains measured for these samples. In both cases, the TT-OSL signals decay rapidly to background and the dose-responses are well represented by a single-saturating exponential function (**Figure 4a, c**). Samples collected from the lowermost units at Galería, i.e., unit GI (ATG10-4), layer TG7 at the base of GIIa (ATG10-9 and ATG10-10) and the base of TZ (ATZ10-4), display distinctly different TT-OSL brightness characteristics. Further details of these differences are provided in the Supporting Information. The dose-saturation properties of the accepted grains permit finite D_e_ determination over very high dose ranges. A pooled distribution of characteristic saturation dose (D_0_) values for the accepted grains of all nine samples is shown in Figure S3 in File S1. Grains with dose-response curves that are best characterised by a single saturating exponential function (*n* = 270) yielded D_0_ values ranging between 250 Gy and 10,000 Gy. Additionally, ∼23% of the accepted grains produced linear dose-response curves over the administered dose range (highest dose-point  = 900–1200 Gy). All of the accepted grains had D_e_ values that are lower than their corresponding 2*D_0_ value; i.e., they are well below the commonly cited threshold for precise burial dose determination.

A representative pIR-IR_225_ decay curve and sensitivity-corrected dose-response curve is shown in **Figure 4e-f**. The pIR-IR_225_ decay curves of these samples typically decrease by >90% within the first 50 s of stimulation and most are optimally fitted with a single saturating exponential plus linear function. All the D_e_ values were obtained from the region of the dose-response that was not in saturation when using this type of fitting function (for example **Figure 4e**).

To examine the potential for athermal loss of K-feldspar pIR-IR signals over burial timescales [73], we performed anomalous fading assessments on subsets of aliquots used to derive D_e_ values. Fading tests were conducted on three to four aliquots per sample for storage times of up to 24 hours [74]. Mean *g*-values normalised to 2 days [75] were calculated from these results, and used to quantify the expected percentage of signal loss per decade of storage time. The mean fading rates for individual samples range between 1.26±0.07 and 1.86±0.22%/decade (**Table 5**) and the combined weighted average *g*-value is 1.39±0.07%/decade. These empirical fading rates are similar to those published previously for pIR-IR_225_ and higher temperature (“pIR-IR_290_”) signals [36], [39], [42], [72], [76]–[80], and agree with pIR-IR *g*-values reported for other Atapuerca karstic infill deposits [47]. Elsewhere, low fading rates (on the order of 1-2%/decade) have been shown to be potential artefacts of laboratory procedures following comparisons made with independent age control, observations of natural signal saturation, and measurements of similarly sized *g*-values for quartz [39], [42], [49]. Consequently, the low *g*-values recorded here are not usually considered indicative of the need for pIR-IR age corrections [39].

**Table 5:** Summary of single-grain TT-OSL and multi-grain pIR-IR_225_ D_e_ values for the Galería samples.

*Luminescence signal*	TT-OSL						pIR-IR_225_						
*Mineral*	Quartz						K-feldspar						
*Resolution*	‘Pseudo’ single-grain						160-grain aliquot						
*Grain size (µm)*	90–125						90–125						
Sample	Accepted/measured	OD (%)^a^	D_e_ (Gy)^b^^,^ c	Weighted skewness value^d^^,^ e	Critical skewness 68% C.I.^d^	Critical skewness 95% C.I.^d^	Accepted/measured	OD (%)^a^	D_e_ (Gy)^f^	*g*-values (%/decade)	Weighted skewness value^d^^,^ e	Critical skewness 68% C.I.^d^	Critical skewness 95% C.I.^d^
AT10-2	33/1000	32±6	591±37 (FMM: *k_2_*)	−0.604	0.426	0.853	6/6	7±3	679±23	1.39±0.04 (n = 4)	0.40	1	2
ATG10-1	46/800	22±5	511±25 (CAM)	−0.007	0.362	0.722	6/6	0±0	658±7	1.43±0.10 (n = 4)	0.06	1	2
ATG10-3	43/800	23±5	572±29 (CAM)	−0.401	0.374	0.747	6/6	2±2	629±8	1.47±0.10 (n = 4)	0.45	1	2
ATG10-7	93/1100	31±4	601±27 (FMM: *k_2_*)	−0.076	0.254	0.508	5/6	6±3	745±23	1.26±0.07 (n = 4)	−0.06	1.1	2.19
ATG10-8	67/1200	20±4	546±21 (CAM)	−0.106	0.299	0.599	6/6	5±2	688±17	1.42±0.16 (n = 4)	0.57	1	2
ATG10-9	17/600	12±11	925±71 (CAM)	−0.730	0.594	1.188	6/6	4±2	929±19	1.51±0.12 (n = 3)	0.94	1	2
ATG10-10	12/700	24±11	813±90 (CAM)	−0.201	0.707	1.414	6/6	6±2	996±27	1.86±0.22 (n = 3)	0.01	1	2
ATZ10-4	21/600	19±8	937±66 (CAM)	−0.133	0.534	1.069	6/6	0±0	1054±15		0.00	1	2
ATZ10-3							6/6	14±5	1131±72		−0.07	1	2
ATG10-4	18/1700	12±9	957±62 (CAM)	−0.744	0.561	1.123							

Also shown are the g-values (fading rates) measured on a subset of aliquots and normalised to a delay time of 2 days.

a OD  =  overdispersion.

b CAM  =  central age model; FMM  =  finite mixture model.

c The FMM was fitted by varying the common overdispersion parameter (σ_k_) between 5 and 30% and incrementally increasing the specified number of k_n_ components. The FMM D_e_ values shown here were obtained from the optimum FMM fit (i.e., the fit with the lowest BIC score; [32]), which corresponded to a σ_k_ value of 15% for both samples (consistent with the lowest empirical overdispersion values obtained for other D_e_ datasets at Galería). Using this approach, the D_e_ datasets of sample AT10-2 and ATG10-7 are shown to contain two and three discrete dose populations, respectively. The main dose components (those containing the majority of individual D_e_ estimates) have been used to derive the final burial doses for these two samples.

d Weighted skewness scores have been calculated on log-transformed D_e_ values using Eq. 7–8 [32]. Critical skewness scores have been calculated using Eq. 16 [125].

e The D_e_ distribution are considered to be significantly positively skewed at the 68% C.I. or 95% C.I. if the weighted skewness value is greater than the corresponding critical skewness value.

f pIR-IR D_e_ values have been calculated using the CAM.

### 2. D_e_ distributions

**Figure 5:**
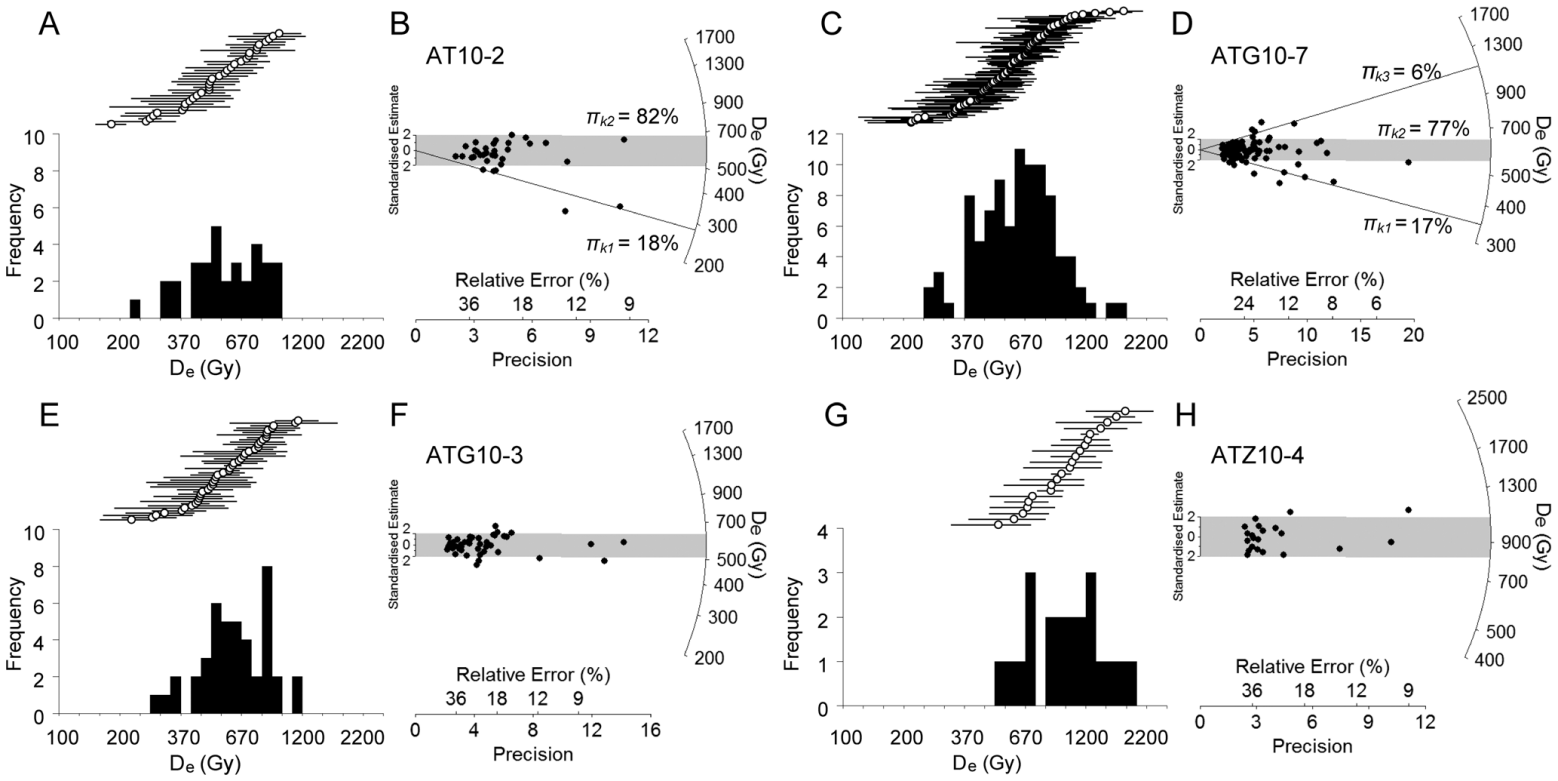
Examples of single-grain TT-OSL D_e_ distributions plotted as histograms (logarithmic x-axis) and radial plots. Data is shown for selected Galería samples. See Figure S4 in File S1 for the D_e_ datasets of all other samples.

The vast majority of Galería samples display similar single-grain TT-OSL D_e_ characteristics (**Table 5**). With the exception of AT10-2 and ATG10-7, all samples have overdispersion values below 25%, which is consistent with values obtained for ideal (well-bleached and unmixed) samples at the neighbouring Atapuerca site of Sima de los Huesos [47]. The log-transformed D_e_ distributions of these seven samples are normally distributed according to published skewness assessments [32] (**Table 5**, **Figure 5** and Figure S4 in File S1) and they do not display pronounced ‘leading-edge’ shapes or elongated high dose tails. The D_e_ datasets are also well-represented by a single dose population, and most of the individual D_e_ values are consistent with the weighted mean burial dose estimate at 2σ (they fall within the grey shaded bands of the radial plots shown in Figures 5F, 5H and Figure S4 in File S1). These observations, together with the relatively high doses measured for these samples, indicate that any D_e_ scatter arising from partial bleaching is likely to be insignificant or effectively absent in these datasets. As such, we have used the weighted mean D_e_ values, calculated using the central age model (CAM) [81], to calculate representative burial dose estimates. The final single-grain TT-OSL D_e_ estimates for these seven Galería samples range between ∼510 Gy and ∼960 Gy (**Table 5**).

Samples AT10-2 and ATG10-7 display more noticeable D_e_ scatter (**Figure 5a-d**) and their overdispersion values (32±6% and 31±4%, respectively) are systematically higher than the weighted mean estimate obtained for the other Galería samples (20±2%) and the ideal Sima de los Huesos samples (21±1%) [47]. Inspection of the radial plots shown in **Figure 5** reveals that sample AT10-2 contains two precise low-dose grains (**Figure 5a**) and that sample ATG10-7 displays a wider spread in D_e_ values at both the lower and upper ends of the D_e_ distribution (**Figure 5d**). For both of these samples, however, the distribution is not positively skewed (**Table 5**). Application of the FMM model [82] confirms that these two D_e_ datasets contain multiple dose populations. The dose components identified by the optimum FMM fit are shown in **Figure 5b and 5d**. These optimum fits were determined according to the BIC criterion [33], using a common overdispersion value of 15% (consistent with the lowest empirical overdispersion values obtained for other D_e_ datasets at Galería). The lowest dose components for the AT10-2 and ATG10-7 datasets yield D_e_ values of 287±34 Gy and 348±35 Gy, respectively (**Figure 5**), and corresponding ages of 123±18 ka and 134±16 ka. These ages are significantly younger than the replicate pIR-IR_225_ ages for AT10-2 and ATG10-7 (**Table 2**). In the case of the latter sample, the age of the low dose component also is not in stratigraphic agreement with the TT-OSL ages obtained on surrounding samples (**Table 2**). The highest dose component identified for sample ATG10-7 contains a very small population of grains (*n* = 6) and its corresponding age (429±71 ka) is not consistent with the associated TT-OSL and IR-IR ages of neighbouring samples (all of which display homogeneous D_e_ distributions). This high dose component is therefore not considered reliable for burial dose determination.

The very young and stratigraphically inconsistent ages obtained for the low dose components of samples AT10-2 and ATG10-7 suggests that partial bleaching is not the primary cause of the above-average overdispersion in these D_e_ datasets. Post-depositional intrusion of younger grains can probably also be ruled out since the host deposits are either capped by well-preserved contacts/speleothems or retain primary bedding features. Beta dose heterogeneity may be a possible cause of the minor, low dose populations identified for AT10-2 and ATG10-7. However, these particular samples were collected from homogenous silt and clay sediments, and were devoid of limestone clasts, gravels or carbonates that could have acted as beta-emitting cold spots or beta-particle attenuators. The minor dose components, which comprise <20% of grains, could therefore be caused by intrinsic sources of D_e_ dispersion, such as grain-to-grain variations in TT-OSL response to fixed measurement conditions or grain-to-grain variations in the thermal stability properties of the TT-OSL signals. For both samples, the main dose components (FMM component 2) contain the vast majority (∼80%) of grains and the resulting TT-OSL ages are in stratigraphic agreement with those obtained for the surrounding samples. We have therefore used these main FMM dose components to derive the final ages for samples AT10-2 and ATG10-7 (**Table 5**).

**Figure 6:**
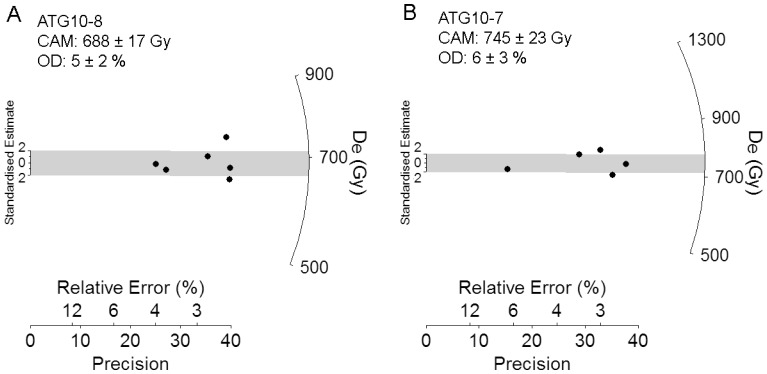
Radial plots showing pIR-IR_225_ D_e_ distributions of representative Galería samples. See Figure S5 in File S1 for the D_e_ datasets of all other samples.

The pIR-IR_225_ D_e_ distributions are shown in **Figure 6** and Figure S5 in File S1. The majority of these D_e_ datasets cover a narrow range and all are normally distributed when assessed using a log weighted skewness test (**Table 5**). The pIR-IR_225_ overdispersion values are generally low (<10% at 1σ; **Table 5**) and are consistent at 2σ with the mean value of 5±1% obtained for well-bleached, unmixed samples elsewhere at Atapuerca [47]. These D_e_ characteristics suggest that any dose dispersion originating from extrinsic or intrinsic sources, including partial bleaching, are relatively insignificant in relation to the size of the measurement uncertainties. As such, we have derived the final pIR-IR_225_ burial doses estimates using the weighted mean (CAM) D_e_ values (**Table 5**).

### 3. Ages

**Figure 7:**
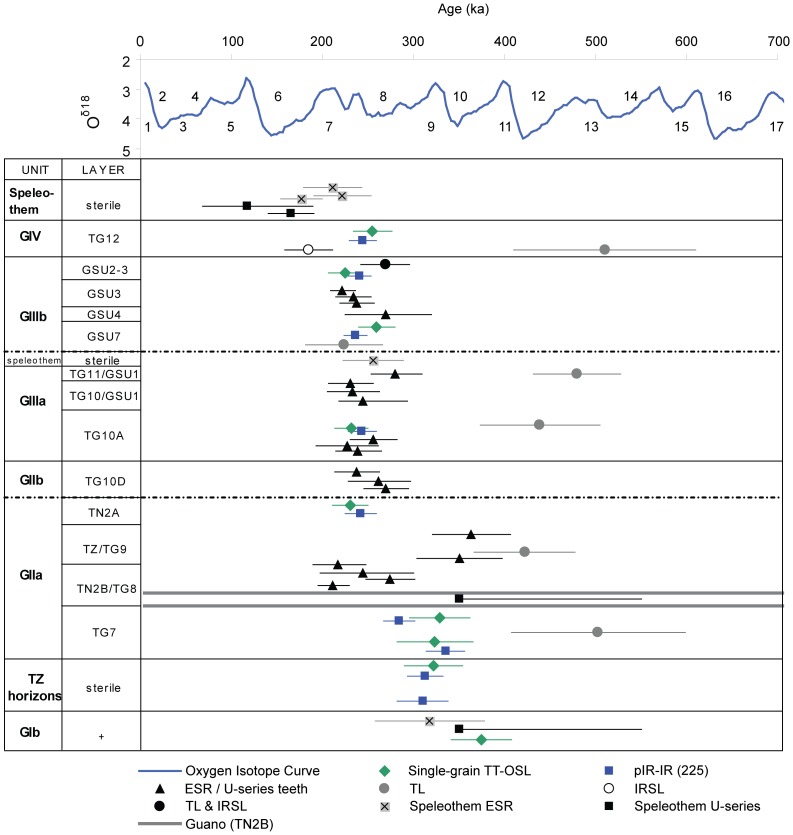
Published ages obtained using different dating methods (including this study) for the various allostratigraphic units and lithologic/archaeo-palaeontological levels at Galería. The data included here has been derived from combined ESR/U-series dating of teeth [15], TL and IRSL dating of sediment [14], ESR dating of calcite/speleothems [11], [104], U-series dating of calcite/speleothems [11], [15]. The numerical age estimates are shown against the Marine Oxygen Isotope curve record [94]. Two additional TL ages of 1340±170 ka (unit GIV) and 3000±1800 ka (unit GI) [14] are not shown on this plot as they lie beyond the x-axis limits (see Table 2 for a full summary of the dataset in [14]).

**Table 2** and **Figure 7** show the final TT-OSL and pIR-IR_225_ ages for the Galería Complex sequence. Both sets of extended-range luminescence ages are stratigraphically consistent and the replicate TT-OSL and pIR-IR_225_ ages of individual samples are in agreement at 1σ. The internal consistency of the two chronological datasets for the entire sequence provides support for the reliability of the TT-OSL and pIR-IR_225_ approaches adopted at this site. The TT-OSL age obtained for sample ATG10-4 suggests that the upper part of GI was deposited 374±32 ka. The two samples collected from the exposure in Zarpazos (ATZ10-3 and ATZ10-4), which are stratigraphically correlated as laying directly below GIIa, produced pIR-IR_225_ and TT-OSL ages of 310–322 ka. The four TT-OSL and pIR-IR_225_ ages for the lowest archaeological level (TG7) range between 284±16 ka and 335±20 ka (samples ATG10-9 and ATG10-10; **Table 2**), and produced a combined weighted mean age of 313±14 ka (*n* = 4) for the first appearance of lithic tools at Galería. Finally, the five samples from the overlying archaeological units (upper GIIa, GIIIa, GIIIb) and sterile GIV horizon (ATG10-8 to AT10-2) produced replicate pIR-IR_225_ and TT-OSL ages ranging between ∼220 and ∼260 ka. These ages are all statistically indistinguishable at 2σ (**Figure 7**), suggesting relatively rapid infilling of the Galería cavity over a timeframe that is comparable to our empirical error ranges. With the possible exception of the ATG10-9 pIR-IR_225_ age, all of the extended-range luminescence ages obtained in this study are in good agreement with the previously published ESR and U-series ages for speleothems [11], [13], [15] and combined ESR/U-series teeth ages [15] at either 1σ or 2σ (**Table 2**, **Figure 7**). The pIR-IR_225_ and TT-OSL ages are also in agreement with the TL and combined TL/IRSL ages obtained on unit GIIIb [14]. However, the other published TL ages for units GIIa, GIIIa and GIV are systematically older than our extended-range luminescence chronologies (**Table 2**, **Figure 7**).

## Discussion

### 1. Suitability of extended-age luminescence techniques in karstic-infill environments

Previous luminescence dating studies of cave deposits have shown that quartz OSL and K-feldspar pIR-IR signals can be completely reset prior to deposition [36], [45], [83], [84]. However, it is less clear whether slower-bleaching TT-OSL signals [50] are able to be sufficiently reset to low levels for sediments that have been transported into caves via alluvial and colluvial processes. To some extent, the adequacy of TT-OSL bleaching in this context is likely to depend on the exposure and recycling histories of grains before they were ultimately washed into the cave. Quartz grains that had experienced prolonged and direct sunlight exposure immediately prior to entrainment and transportation into the cave and/or experienced relatively short residency times in well-bleached transient sediment bodies may still be amenable for TT-OSL dating, in spite of the unfavourable bleaching conditions they potentially experience during their incorporation into the cave deposit. Samples from the nearby Sima de los Huesos site, which is part of the same karst system, display seemingly well-bleached single-grain TT-OSL D_e_ distributions (i.e., low overdispersion values, single dose populations, non-skewed D_e_ distributions) [47]. The resultant weighted mean TT-OSL ages for these samples are also in good agreement with independent chronologies established at the site using U-series analysis [85]. In the same study it was shown that surface deposits located near the cave entrance have a very low mean TT-OSL residual of ∼7 Gy (<1% of the mean D_e_ value for the Sima de los Huesos samples), which is similar to the lowest values measured elsewhere for modern-age aeolian samples [37], [51], [52], [71]. This modern sample provides a useful analogue for the potentially well-bleached nature of the sediments found preserved in the cave environment. The majority of single-grain TT-OSL D_e_ distributions obtained in the present study are consistent with those reported for the Sima samples and the resultant TT-OSL ages are also in agreement with existing ESR, U-series and combined ESR/U-series chronologies. Together, the Galería Complex and Sima de los Huesos TT-OSL results indicate that the sediments blanketing the surrounding hills of the Atapuerca karst complex (i.e., the external sources of the karstic infill sediments) probably experienced prolonged bleaching periods (in the order of weeks to months) prior to entering the cave, as suggested previously [14]. At Galería, grains from units GIIa (upper) to GIV are also likely to have experienced additional sunlight exposure within the cave chamber prior to burial owing to their proximity to the nearby cave entrance (TN shaft).

Encouragingly, the sediments that were not directly sourced from the nearby cave opening (unit GI and the Zarpazos deposits, i.e., those transported through the endokarst system) yielded TT-OSL ages in agreement with replicate pIR-IR ages and the existing ESR and U-series (minimum) ages established on bracketing speleothems (**Figure 7**). Unit GIb was initially considered to be autochthonous to the cave system [12], [57] and possibly sourced from dissolution of the host limestone bedrock. However, this interpretation is not consistent with the reasonable TT-OSL chronologies we obtain for samples ATG10-4, ATZ10-3 and ATZ10-4. If the dated grains had been directly derived from weathered bedrock, it is likely that they would have yielded either “infinite” D_e_ values or stratigraphically incoherent ages, as well as disagreement with replicate pIR-IR ages. Moreover, truly autochthonous sediments from this karst system are unlikely to have yielded the quantities of coarse-grained quartz grains found in our samples. Dissolution of limestone fragments collected from the Galería site as part of the present study showed that the Cretaceous bedrock contains very limited concentrations of quartz (<1%; [86]) and that the available quartz is exclusively <50 µm. In contrast, the luminescence dating samples collected from Zarpazos and unit GIb at Galería are composed primarily of coarse sand and silt grains that are >60 µm in size and the mineralogies are strongly dominated by quartz [14], [86]. These observations, together with the consistency of the resultant luminescence dating results, suggest that the original sediment source was most likely external to the karst system.

The samples from unit GIb in Galería and Zarpazos also display distinctly different quartz luminescence brightness properties in comparison with the overlying samples (units upper GIIa to GIV; see File S1), indicating a potentially different sedimentary origin or geomorphic reworking history. This finding is consistent with the lack of exokarstic material (i.e., large mammal remains) in unit GIb and suggests that, at the time of deposition, the cave was closed-off from the immediate surrounding, and that these sediments were derived from an external source located some distance away. Consequently, it would be inappropriate to assume that sediments from GIb were fully bleached immediately prior to their final deposition, as was demonstrated [47] for sediments sourced from the immediate cave entrance (GII-GIV). In this regard, the TT-OSL and pIR-IR ages obtained for samples ATG10-4, ATZ10-3 and ATZ10-4 could conservatively equate to maximum age estimates for the emplacement of the host deposits at Galería. The similarity between the TT-OSL ages and the bracketing (minimum) ages of associated speleothems suggests that the sands of Zarpazos and unit GI were deposited relatively quickly after their initial incorporation into the karst system (i.e., over a time span similar to our analytical uncertainties). However, further luminescence studies are needed on similar “internal” facies to better constrain transportation times within the deeper cave system.

In assessing the suitability of the TT-OSL and pIR-IR chronologies obtained in this study, it is worth considering how our ages might change if we were to apply additional corrections for potential athermal and thermal signal losses over burial timescales. The need for application of a TT-OSL thermal instability correction is not clear-cut from existing known-age field assessments (see synthesis in [87]), though laboratory lifetime predictions suggest that an empirical correction may be prudent for some samples at least, particularly when dating over Early Pleistocene timescales. In the present context, it is also not known whether existing (multi-grain aliquot) laboratory lifetime predictions are of direct relevance to the specific grain populations isolated in our single-grain analysis. Nevertheless, if we assume a worst-case scenario and apply a first order TT-OSL thermal stability correction based on published laboratory lifetime calculations [48] and a sample burial temperature of 10°C (based on the mean current annual air temperature recorded inside the deeper chambers of the Atapuerca karst system [88] and outside the cave at the nearby Villafría (INM-2331) weather station), the TT-OSL ages of the Galería samples increase by only 6–16 ka. The difference between the original and corrected TT-OSL ages equate to <4% and is statistically insignificant at 1σ for all samples. Any potential systematic biases arising from signal instability are therefore largely inconsequential over the Middle Pleistocene timescales considered here.

The application of an anomalous fading correction to our pIR-IR_225_ ages (based on the empirically determined g-values) causes the resultant chronologies to increase by 12–20% but none of the ages change beyond their original 2σ error ranges. Importantly, the fading corrected pIR-IR_225_ ages are still in broad agreement with existing ESR, U-series and combined ESR/U-series chronologies at 2σ and they are still systematically younger than the TL ages [14] for units GIIa, GIIIa and GIV. Hence, our overall findings remain unchanged regardless of whether we opt for the original or fading corrected pIR-IR_225_ ages.

### 2. Comparison with previous luminescence dating results

As already noted, the majority of polymineral fine-grain TL ages [14] are significantly older than the chronologies established using other dating approaches, as well as those reported here (**Figure 7** and **Table 2**). Only the TL ages of 269±27 ka and 224±42 ka obtained for TG11 (upper GIIIb) are in agreement with our TT-OSL and pIR-IR ages at 1σ. The TL age of 503±95 ka for sublevel TG7 of lower GIIa is systematically older than the four ages obtained for this same unit using TT-OSL and pIR-IR_225_ (mean age 313±14 ka); though this TL age is in agreement with our ages at 2σ owing to its large relative uncertainty (19%). Given the systematic differences with the TL results, it seems plausible that this technique may not have provided representative burial ages for the Galería sequence. The most likely sources of inaccuracy with the TL ages include problems of partial bleaching, the use of 2π- instead of 4π-geometry gamma spectrometry in heterogeneous sedimentary matrices, and limitations with the chosen D_e_ measurement and data analysis procedures. TL dating was performed at Galería using a multiple-aliquot additive-dose (MAAD), total-bleach method, for which tens of thousands of grains were measured simultaneously and a single D_e_ value was computed per sample via extrapolation of the dose-response curve [89]. Though our single-grain TT-OSL results suggest that these samples were exposed to prolonged sunlight before transportation and burial, the adequacy of TL signal bleaching was not specifically tested in the earlier study [14]. Indeed, the TL plateau tests presented for some samples showed an increase in D_e_ with temperature (see Figure 6 and S2 of [14]), which may indicate insufficient bleaching of deeper TL traps. Though IRSL residual doses were shown to be very low for nearby surface sediments, the same assessments were not performed on the TL signals; hence the likelihood of MAAD TL resetting remains unknown in this environment. Additional D_e_ inaccuracies could have been introduced by the use of poorly defined dose-response curve fits coupled with significant extrapolation of the MAAD responses [89], [90], particularly as many of the measured dose-response curves lay within 50% of saturation [91]. In light of these analytical uncertainties and the systematic TL age offsets seen throughout the sequence (**Figure 7**), we consider that the TL chronologies developed for Galería, and by extension those from other Atapuerca sites (e.g., TD10 Gran Dolina), should be viewed with a degree of caution (see also [92]).

It is also worthwhile noting that five additional IRSL ages were not reported [14] because they diverged from replicate TL ages at Galería and they ceased to increase with stratigraphic depth beyond ∼250 ka. These trends were taken to be indicative of IRSL signal saturation but a similar non-incremental age-depth profile is apparent for both the ESR/U-series dataset [15] (GIIb to GIIIb) and for our extended-range luminescence chronologies (units upper GIIa to GIV). The similarities between these independent geochronological results suggests that the allochthonous Galería sequence genuinely accumulated over a relatively short timescale and thus the rejected IRSL ages [14] may have been more reliable than originally thought.

### 3. Chronostratigraphic implications of the new extended-range luminescence ages and comparison with recent ESR/U-series dating of teeth

Our new TT-OSL and pIR-IR results enable us to quantitatively evaluate the chronostratigraphic model proposed previously for the Galería sequence [16]. According to micromorphological data and pollen analysis undertaken on units GII to GIII, the allochthonous Galería sequence records a climatic deterioration from warm and wet condition (GIIa) to a cool and temperate phase (GIII-GIV) [9], [62], [93], and may correspond to a single interglacial–glacial cycle [8], [10]. The upper section of the preceding sterile level (GIb) also records interglacial-like conditions characterised by high biological activity, degradation and bioturbation [9], [12], [16], [57]. On the basis of these palaeoenvironmental indicators, it was suggested that (i) the beginning of the Galería sequence (GIb) accumulated during either MIS 9 or MIS 11, (ii) the lower archaeo-palaeontological levels (GIIa) formed within MIS 9, and (iii) the upper units (GIIb-GIIIb) were deposited during MIS 8 and possibly early MIS 7. The TT-OSL age for GIb (374±33 ka) aligns closely with the MIS 11/MIS 10 boundary but is also consistent with an MIS 9 age at 2σ. The mean luminescence age for the base of GIIa is 313±14 ka (i.e., TG7; *n* = 4) and indicates that this horizon accumulated during MIS 9. Together, our TT-OSL ages suggest that levels GIb and GIIa were either deposited continuously from early MIS 11/late MIS 10 through to MIS 9, or, if we consider the intervening finite stalagmite age (318±60 ka; **Table 2**) and the relatively warm conditions recorded in GIb, that both levels were deposited over a shorter time period within MIS 9. The shared sedimentological properties of the lower GIIa horizons and the upper part of unit GI [16] suggests that the two depositional episodes could have occurred during a closely spaced time period (i.e., within the same interglacial cycle), hence the latter interpretation may be the most feasible.

Our numerical dating results provide broad support for earlier chronostratigraphic interpretations [16]. We note, however, that the age of unit GIb (sample ATG10-4) is based on a relatively low number of TT-OSL D_e_ estimates. Further single-grain TT-OSL measurements would ideally be needed to improve the precision (and potentially accuracy) of this particular age estimate and to better constrain the chronological relationship between GIb and the base of GII. Regardless, we do not observe any evidence to suggest that the base of the archaeo-palaeontological levels at Galería Complex is ∼500 ka (MIS 13), as indicated by the earlier TL dating results. Instead, our combined TT-OSL and pIR-IR datasets reveal that the earliest Acheulean lithics at Galería (i.e., those recorded in level TG7) were most likely formed during MIS 9 (i.e., 337–300 ka; [94]).

The 20 ESR/U-series teeth ages [15] reveal a potential temporal discontinuity between sub-units GIIa and GIIb at Galería (**Figure 7**). This chronological divergence occurs between archaeo-palaeontological levels TG10D and TG9, and is marked by an increase in age from 237–269 ka (*n* = 4) to 350–363 ka (*n* = 2) (see Figure 11 of [15]). It is worth highlighting that this apparent shift in the ESR/U-series ages is potentially negated by four additional teeth ages of 211–270 ka obtained on the underlying TG8 horizon of GIIa. These four ages were deemed unreliable on geochemical grounds [15], though it was acknowledged that there is no direct evidence for post-depositional uranium leaching in at least three of these specimens. A similar chronological shift is apparent in our luminescence dataset; however this potentially occurs at a lower stratigraphic position, between levels TG7 and TN2A of unit GIIa (**Figure 7**). This temporal discontinuity in unit GIIa appears to coincide with a major alteration in the local depositional environment at Galería, as evidenced by a change in the intrinsic TT-OSL characteristics of samples collected from TN2A upwards. The increased frequency of TT-OSL-producing grains and higher signal sensitivities observed for samples ATG10-8 through to AT10-2 (Figure S1 in File S1 and Table S3 in File S1) indicates an apparent shift in local sediment sourcing in comparison with units TG7, lower Zarpazos and GI. These changes in luminescence characteristics are consistent with lithostratigraphic evidence from unit GIIa, which suggest that the Galería chamber was opened-up to the cave exterior at this stage and started to be filled by large quantities of locally derived terra rossa sediment. The field-based evidence for a major change in sedimentary regime includes (i) the presence of large limestone blocks above layer TG7 at TN, which indicates extensive roof collapse at the southern end of the chamber; (ii) the appearance and increasing dominance of exokarstic material in layers TN2B, TG8 and TG9, as documented by the micro-morphological, microfacies and lithological analysis [9], [16], [62]; (iii) the deposition of a large guano layer and evidence of peat formation [62] immediately above layer TG7; and (iv) the occurrence of speleothem formations in layer TG8, which lies between levels TG7 and TN2A. Both the guano layer and the speleothem formation are indicative of a potential hiatus in sediment deposition prior to the cave opening event. The new TT-OSL and pIR-IR_225_ ages suggest that this depositional hiatus occurred between 313±14 ka (the mean age of TG7) and the opening of the cave chamber at (or slightly before) ∼250 ka (the age of TN2A).

In comparing the luminescence ages from layer TN2A (sample ATG10-8) with the two combined ESR/U-series ages from levels TZ and TG9 (samples TZ02-02 and TG02-06), it is worth bearing in mind that the lateral associations between these horizons is somewhat ambiguous. Level TN2A is only preserved in the southern section of the profile, whereas level TG9 is found exclusively in the northern sector. It has been reported that TN2A is either located above or is correlative with level TG9 [59], and that both layers lie above TG8. It has also been acknowledged, however, that the true relationship between these levels is not easy to distinguish because they are laterally discontinuous and have undergone some post-depositional alterations [59]. Furthermore, one of the herbivore teeth dated by ESR/U-series was obtained from the entrance of the TZ chamber, which is disconnected from the TN section and is not likely to be directly representative of the GIIa levels preserved ∼10 m away at the southern end of the Galería exposure. Added to this complexity is the fact that the luminescence and ESR/U-series samples were collected from sections that accumulated through different sediment conduits (TZ and TN, respectively). The difference in the ages obtained for TG9 and TN2A suggests that these deposits are not directly correlative, and they may have been deposited non-synchronously. Resolving the complex stratigraphic relationships observed in sub-unit GIIa will require a higher resolution dating study using complementary methods, as well as additional, quantitative sedimentological analyses.

It is interesting to compare the new Galería ages with those obtained for other Middle Pleistocene infill sequences at Atapuerca, namely Gran Dolina level TD10, the uppermost units of Sima del Elefante and the clay breccia infills at Sima de los Huesos. The TD10 level of Gran Dolina (located 30 m away) contains an abundant Mode II Acheulean assemblage and has been dated to between 337±29 ka and 418±63 ka using combined ESR/U-series analysis of teeth [95] (although see below for further discussion on contradictory TL/IRSL ages by [14]). The new weighted mean luminescence age of 313±14 ka for the lowermost archaeo-palaeontolgical level at Galería (TG7) may be contemporaneous with the age range of the TD10 sub-levels (TD10.1 and TD10.2), indicating that the two sites could have been occupied broadly simultaneously or over closely spaced time frames. Similarly, our ages for the earliest hominin presence at Galería appear to be correlative with the deposition of Upper Red Unit TE19 at the nearby site of Sima del Elefante, which contain sparse Mode II lithics and has been dated to 350–200 ka using biochronological associations [96]. Recently, new luminescence ages were presented [47] for the Sima de los Huesos “café con leche” bone breccia found directly above a rich *H. heidelbergensis* fossil assemblage and a single, well-shaped Acheulean hand-axe. The TT-OSL and pIR-IR_225_ mean age for this bone breccia is 427±12 ka (*n* = 6), which provides a firm minimum age for the hominin fossils. The combined luminescence chronology for the Sima de los Huesos assemblage reveals that there is no temporal overlap with the archaeological layers at Galería; the evidence for hominin presence at these two sites is separated by at least ∼100 ka.

The new luminescence ages for Galería units GII and GIII confirm the presence of the Acheulean tradition in north-central Iberia within MIS 9 and MIS 8, at a time when Middle Palaeolithic technologies were appearing elsewhere in western Europe (Organc 3 [97], [98]; see [99] for overview). Our chronological results therefore support previous evidence pointing to the co-existence of these two lithic traditions during the Middle-Pleistocene [99]–[103]. Interestingly, the presence of Levallois-like methods at the Gran Dolina TD10.1 (upper) sub-level, as well as the progressive decrease in the number of large cutting tools within the entire 4 m-thick TD10 level, have been interpreted as representing a local Mode 2 to Mode 3 transitional phase [6] (similar observations have also been reported for the ‘top of Galería’ [19], albeit with limited information regarding tool description and their exact stratigraphic position). ESR/U-series dating of teeth from TD10.1 places this local transition [6] at ∼350 ka (337±29 ka and 379±57 ka; [5], [95]), but younger ages of ∼240 ka have been obtained for bracketing layers TD11 and TD10.2 using polymineral fine-grain IRSL and TL dating, respectively [14]. The ESR/U-series ages for TD10.1 clearly pre-date our new ages and the published ESR/U-series ages [15] for units GIIb and GIII, which contain Mode II Acheulean artefacts. If the ESR/U-series ages for TD10.1 are correct, then there is an apparent temporal contradiction between the TD10.1 and Galería lithic records. New radiometric dating efforts at Gran Dolina, which are currently underway, will prove crucial for resolving this discrepancy, and will lead to an improved understanding of the pattern of technological change that occurred at Atapuerca during the Middle Pleistocene.

## Conclusion

The extended-range luminescence chronologies obtained in this study enable us to refine and extend the existing numerical age constraint at the Middle Pleistocene archaeological site of Galería. Our combined TT-OSL and pIR-IR ages reveal that the basal sterile sands (unit GIb) underlying the archaeo-palaeontological sequence were deposited ∼370 ka. The first hominin exploitation of Galería appears to have taken place during MIS 9 at ∼313 ka, as recorded by the accumulation of Mode II lithics in layer TG7 of sub-unit GIIa. This period of hominin activity occurred prior to the opening of the main TN shaft. The new luminescence ages for horizon TN2A, which overlies the organomineral and speleothem formations (TG8), suggest that the main chamber became directly exposed to the exterior by ∼240 ka. Hominin exploitation of animal carcasses (pitfall trap remains) continued during MIS 8 and early MIS 7 and was accompanying by a relatively rapid sediment infilling phase, as suggested by the indistinguishable ages obtained for the upper part of unit GIIa to unit GIV.

The new ages for the Galería sequence also provide insight into the origin of chronological disagreements published previously using different radiometric dating methods. The TT-OSL and pIR-IR ages obtained in this study are in overall agreement with the published ESR and U-series speleothems ages and combined ESR/U-series teeth ages at 2σ. However, the published TL ages for units GIIa, GIIIa and GIV are systematically older than both our luminescence chronologies and the ages obtained using other dating techniques (by ∼200 ka in most instances). Given the significant offset with these TL ages, we suggest that chronologies established elsewhere at Atapuerca using this approach should be interpreted with caution.

The suitability of the extended-range chronologies presented in this study is supported by laboratory quality assurance tests (dose-recovery tests), assessments of single-grain D_e_ datasets, agreement between replicate TT-OSL and pIR-IR_225_ ages, and the stratigraphic consistency of the resultant chronologies. Our data are consistent with the recent results presented for the neighbouring site of Sima de los Huesos [47] and suggest that TT-OSL and pIR-IR techniques offer good potential for constraining human occupation and depositional histories at the Middle Pleistocene Atapuerca sites. We emphasise, however, that the applicability of these extended-range luminescence dating techniques may vary at other localities and that sample-specific suitability assessments should always be performed in pIR-IR and TT-OSL dating studies. This study also demonstrates the potential for using luminescence sensitivity characteristics as a proxy for identifying changes in local depositional conditions and sediment sourcing. This novel approach may provide useful ancillary information for lithostratigraphic assessments of autochthonous versus allochthonous infill horizons at other Atapuerca sites.

## Supporting Information

File S1 **This file contains additional text about methodology and results, as well as associated figures and tables.** Explanations are provided about measurement conditions and data analysis used for both the TT-OSL and pIR-IR_225_ signals (e.g., D_e_ calculation and rejection criteria). Dose recovery test results for the pIR-IR and the TT-OSL signals are shown in Table S4. File S1 also contains the TT-OSL signal brightness characteristics and single-grain rejection statistics obtained for all samples. This file also contains Table S1-S5 and Figure S1-S5. Table S1, Details of the Galería luminescence dating samples analysed in this study. Table S2, Radionuclide activities (Bq kg-1) and daughter-to-parent ratios obtained from high resolution gamma spectrometry (HRGS) measurements of the 238U and 232Th decay chains. Table S3, Single-grain TT-OSL classification statistics for the Galería samples. Table S4, Results obtained for the dose recovery tests performed using protocol A of Table 4 for the TT-OSL signal. Table S5, Rejection statistics from the single-grain TT-OSL dose recovery test performed on samples ATG10-3 corresponding to Table S4. Figure S1, (A) Cumulative light-sum plots for the Galería samples constructed from the ranked net natural test dose signal (Tn) (using the first 0.24 s of laser stimulation minus a background-subtraction from the final 0.25 s). Plot (B) shows ranked signal counts normalised to the given Tn dose (200–300 Gy). Data shown is for single-grain TT-OSL measurements made using the 90–125 µm grain fraction (∼18 grains per hole; Arnold et al., 2012). Figure S2, Single-grain TT-OSL dose recovery test results (∼18 grains per hole). Figure S3, Histogram showing the distribution of single-grain TT-OSL D0 values for the Galería samples. Figure S4, Single-grain TT-OSL De distributions for the Galería samples, shown as histograms (left column) and radial plots (right column). Figure S5, Radial plots showing the pIR-IR225 De distributions of the remaining Galería samples not included in Figure 6 of the main text.
(DOC)
