# Supplementary material for: New Luminescence Ages for the Galería Complex Archaeological Site: Resolving Chronological Uncertainties on the Acheulean Record of the Sierra de Atapuerca, Northern Spain
Source: PLoS One. 2014 Oct 22;9(10):e110169. doi: 10.1371/journal.pone.0110169 (PMC4206284; doi:10.1371/journal.pone.0110169)
Supplement: File S1 — This file contains additional text about methodology and results, as well as associated figures and tables. Explanations are provided about measurement conditions and data analysis used for both the TT-OSL and pIR-IR225 signals (e.g., De calculation and rejection criteria). Dose recovery test results for the pIR-IR and the TT-OSL signals are shown in Table S4. File S1 also contains the TT-OSL signal brightness characteristics and single-grain rejection statistics obtained for all samples. This file also contains Table S1-S5 and Figure S1-S5. Table S1, Details of the Galería luminescence dating samples analysed in this study. Table S2, Radionuclide activities (Bq kg-1) and daughter-to-parent ratios obtained from high resolution gamma spectrometry (HRGS) measurements of the 238U and 232Th decay chains. Table S3, Single-grain TT-OSL classification statistics for the Galería samples. Table S4, Results obtained for the dose recovery tests performed using protocol A of Table 4 for the TT-OSL signal. Table S5, Rejection statistics from the single-grain TT-OSL dose recovery test performed on samples ATG10-3 corresponding to Table S4. Figure S1, (A) Cumulative light-sum plots for the Galería samples constructed from the ranked net natural test dose signal (Tn) (using the first 0.24 s of laser stimulation minus a background-subtraction from the final 0.25 s). Plot (B) shows ranked signal counts normalised to the given Tn dose (200–300 Gy). Data shown is for single-grain TT-OSL measurements made using the 90–125 µm grain fraction (∼18 grains per hole; Arnold et al., 2012). Figure S2, Single-grain TT-OSL dose recovery test results (∼18 grains per hole). Figure S3, Histogram showing the distribution of single-grain TT-OSL D0 values for the Galería samples. Figure S4, Single-grain TT-OSL De distributions for the Galería samples, shown as histograms (left column) and radial plots (right column). Figure S5, Radial plots showing the pIR-IR225 De distributions of the remaining Galerí [file pone.0110169.s001.doc]

**Supporting Information**

***Single-grain TT-OSL measurements***

For these measurements, 90-125 μm quartz grains were loaded onto single-grain aluminium discs drilled with 300μm × 300μm holes. It is estimated that ~18 grains were placed into each hole using this configuration (Arnold et al., 2012). However, we are confident that true single-grain resolution has been maintained in this study because of the low frequency of grain-hole positions (let alone individual grains) that produced TT-OSL signals. On average, 74% of measured grain-hole positions yielded TT-OSL signals that were statistically indistinguishable from background levels for the nine Galería samples measured (**Table S3**, **Figure S1**). Demuro et al. (2013) have shown that samples characterised by such low yields (<30%) of luminescent grains are not likely to exhibit significant averaging effects when measuring ~18 grains per hole. We have therefore used this measurement to boost the number of usable grains per disc while minimising any 'pseudo' single-grain averaging effects.

***Equivalent dose (D_e_) calculation and Rejection criteria***

The TT-OSL single-grain D_e_ estimates were calculated from the first 0.24 s of stimulation after subtracting a late-light background count obtained from the last 0.26 s of stimulation. The SAR dose-response curves were fitted using either a linear or a single-saturating exponential function. Single-grain TT-OSL D_e_ estimates were rejected if: (1) the luminescence signal of the natural test dose, T_n_, was not >3σ above the late-light background signal; (2) the net T_n_ signal had a relative error of >30%; (3) the recycling ratio (i.e., sensitivity-corrected luminescence responses (L_x_/T_x_) for two identical regenerative doses) was not consistent with unity at 2σ; (4) the recuperation ratio, calculated as the ratio of the sensitivity-corrected 0 Gy dose point (L_0_/T_x_) to the sensitivity-corrected natural (L_n_/T_n_), was >5%; (5) the OSL IR depletion ratio (measured separately using two conventional single-grain OSL SAR cycles; Duller, 2003) was less than unity at 2σ; (6) the sensitivity-corrected natural signal (L_n_/T_n_) did not intercept the sensitivity-corrected dose-response curve; (7) the dose-response curve displayed anomalous properties (e.g., zero or negative responses with increasing dose) and/or poor Monte Carlo fits; (8) the L_n_/T_n_ value intercepted the saturated part of the dose-response curve; and (9) the relative error on the calculated D_e_ was >50%. Single-grain TT-OSL rejection statistics for the measured Galería samples are shown in **Table S3**.

Multi-grain K-feldspars D_e_ values were calculated from the first 4 s of stimulation after subtracting a mean background count from the last 10 s of stimulation. The pIR-IR dose response curves were fitted using either a single-saturating exponential or a saturating-exponential plus linear function. D_e_ estimates were rejected if: (1) the recycling ratio (i.e., sensitivity-corrected luminescence responses (L_x_/T_x_) for two identical regenerative doses) was not consistent with unity at 2σ; (2) the recuperation ratio, calculated as the ratio of the sensitivity-corrected 0 Gy dose point (L_0_/T_x_) to the sensitivity-corrected natural (L_n_/T_n_), was >5%.

***pIR-IR dose recovery tests***

To determine the most suitable pIR-IR SAR measurement and preheat conditions for the Galería samples we undertook dose recovery tests on ~360-grain aliquots of K-feldspar from samples ATG10-3 and ATG10-10. For each sample, two batches of aliquots were bleached in the SOL2 simulator for 1.5 hours. A laboratory dose of 600 Gy (ATG10-3) or 1000 Gy (ATG10-10) was administered to the first batch of aliquots prior to undertaking the SAR D_e_ measurements. The second batch of bleached aliquots was measured without any prior dosing to determine an appropriate residual dose subtraction. Three different pIR-IR SAR protocols were tested in these experiments: a pIR-IR_225_ protocol involving pIR-IR stimulation temperatures of 225°C and regenerative- / test-dose preheat temperatures of 250°C for 60 s; a pIR-IR_255_ protocol involving pIR-IR stimulation temperatures of 255°C and preheat temperatures of 280°C for 60 s (ATG10-10 only); and a pIR-IR_290_ protocol involving pIR-IR stimulation temperatures of 290°C and preheat temperatures of 320°C of 60 s (**Table S4**). For sample ATG10-3, we were able to recover the given dose accurately with the pIR-IR_225_ protocol (recovery ratio = 1.00 ± 0.03). The pIR-IR_290_ protocol systematically overestimated the given dose for this sample, though the net recovered dose ratio is consistent with unity at 2σ (ratio = 1.09 ± 0.05). In the case of sample ATG10-10, the pIR-IR_225_ protocol again produced net recovered to given dose ratios in agreement with unity at 1σ (recovery ratio = 0.98 ± 0.03), whereas the pIR-IR_255_ and the pIR-IR_290_ protocols significantly overestimated the given dose (recovery ratios = 1.17 ± 0.04 and 1.13 ± 0.06, respectively). These results reveal that the more stringent pIR-IR stimulation and preheating temperatures are potentially unsuitable for dating the Galería samples, which is consistent with results obtained elsewhere at Atapuerca sites (Arnold et al., 2014, submitted). On the basis of these dose-recovery assessments, we have employed the pIR-IR_225_ protocol for dating purposes.

***TT-OSL dose recovery tests – multi-grain aliquots***

Two types of multi-grain TT-OSL dose-recovery tests were performed on sample ATG10-3. In the first experiment, a batch of six ~4400-grain aliquots were prepared and placed under direct sunlight for a period of 3.5 weeks (Burgos, during May-June 2013). Four of these aliquots were given a dose of 550 Gy, while the rest were left untreated. The D_e_ value were then measured using a multi-grain aliquot version of SAR protocol A shown in **Table 4** (replacing single-grain laser stimulations with blue LED OSL stimulations at 125^o^C for 100 s in steps 3, 5, 9 and 11). Dose recovery ratios were calculated by subtracting the weighted mean residual D_e_ of the non-dosed aliquots from the weighted mean D_e_ of the dosed aliquots. Five of the six measured aliquots displayed recycling ratios within 2σ of unity and recuperation ratios of below 5% (not shown), favouring the general applicability of the TT-OSL protocol. However, the net recovered dose of the sun-bleached aliquots overestimates the given dose by 20% (1.21± 0.06; **Table S4**).

For the second experiment, nine aliquots were prepared and measured in the same way but none of these aliquots were bleached under sunlight prior to D_e_ determination (i.e., the natural signals were left intact for both the dosed and non-dosed sub-sets of aliquots). After subtracting the weighted mean natural dose of the non-dosed aliquots (*n* = 4) from that of aliquots that had been given a dose (*n* = 5), we obtained a recovered to given dose ratio in agreement with unity at 1σ (0.98 ± 11; **Table S4**). It was therefore possible to recover a known dose from aliquots that had not experienced prior bleaching of their natural signals. The reason for the different results between the two experiments is unclear and will be the subject of future research. It is possible that certain grain types from these samples may undergo large sensitivity changes as a result of being subjected to sunlight bleaching and immediate dosing using a strong laboratory beta source. Such experimental conditions do not necessarily reflect the natural bleaching and dosing histories experienced by these grains and may therefore be producing unrepresentative dose-recovery results in this instance. The ability to successfully recover a dose on unbleached aliquots provides us with greater confidence in the suitability of the TT-OSL SAR protocol for our samples.

***TT-OSL dose recovery tests – single grains***

A subsample of etched 90-125 quartz grains of sample ATG10-3 were placed under direct sunlight for 6 weeks (Burgos, during July-August 2013) for single-grain TT-OSL dose-recovery experiments. A fraction of these grains was given a dose of ~554 Gy and measured using protocol A of **Table 4**. The remaining bleached grains were measured without any prior dosing to determine an appropriate residual dose. The D_e_ distributions for the non-dosed and dosed sun-bleached grains are shown in **Figure S2a-b**. The majority of the TT-OSL residual doses range between 1 and 130 Gy, and the overdispersion of the resultant D_e_ distribution is 55%. The D_e_ values of the dosed grains are normally distributed and range between ~280 Gy and ~1080 Gy, with an overdispersion value of 9 ± 5% (**Table S4**). One third of the non-dosed grains (35%) have residuals that are within 2σ of zero, and approximately one quarter (24%) produced residuals that are within 2σ of <20 Gy. The wide spread in the measured residual doses suggests that there are significant grain-dependent variations in TT-OSL signal bleaching rates. In spite of the spread in residual dose estimates, the weighted mean D_e_ value produces a reasonable fit for the radial plot data (grey bar in **Figure S2b**) and is therefore considered suitable for calculating the net recovered dose ratio. The weighted mean D_e_ values for the residual and dosed grains populations are 92 ± 3 Gy and 696 ± 23 Gy, respectively (**Table S4)** The resulting weighted mean recovered to given dose ratio is within 2σ of unity (**Table S4**), supporting the suitability of the single-grain TT-OSL protocol. The results of the single-grain and multi-grain aliquot dose-recovery tests therefore differ when using prior sunlight bleaching. This may be explained by the exclusion of less suitable grain types and the avoidance of deleterious multi-grain averaging effects at the single-grain scale of analysis; but further work is needed to ascertain this definitively. Regardless, the single-grain dose-recovery test results provide general support for the suitability of the SAR protocol adopted in the present study.

***Single-grain TT-OSL signal brightness characteristics***

The single-grain measurements reveal that a relative low proportion (15-40%) of grain-hole positions produce statistically significant TT-OSL signals (i.e., T_n_ signal intensities that are >3σ above background for a test dose of 200-300 Gy) (**Figure S1a**) and only 0.2-2.3% of measured holes produce signals >1 net counts/Gy/0.24 s (**Figure S1b**). There is some inter-sample variation in grain brightness characteristics, with between 10 and 30% of the measured grain-holes producing 90% of the combined luminescence signal per sample (**Figure S1a**). Samples collected from the lowermost units at Galería, i.e., unit GI (ATG10-4), layer TG7 at the base of GIIa (ATG10-9 and ATG10-10) and the base of TZ (ATZ10-4), display distinctly different grain brightness characteristics. Specifically, fewer of the measured grain-hole positions produce TT-OSL for these samples, and the luminescent grains appear to display more uniform and dimmer signals (**Figure S1a-b**). Analysis of the single-grain rejection statistics (**Table S3**) reveals that these four samples also contain lower proportions of grains in all categories, including a lower percentage of accepted grains (1.1-3.5%) when compared with samples from the overlying units (3.3-8.5%).

***References***

Arnold, L.J., Demuro, M., Navazo Ruiz, M., 2012. Empirical insights into multi-grain averaging effects from ‘pseudo’ single-grain OSL measurements. Radiation Measurements 47, 652–658.

Arnold, L.J., Demuro, M., Parés, J.M., Arsuaga, J.L., Aranburu, A., 2014. Luminescence dating and palaeomagnetic age constraint on hominins from Sima de los Huesos, Atapuerca, Spain. Journal of Human Evolution 67, 85–107.

Arnold, L.J., Demuro, M., Parés, J.M., Pérez-González, A., Arsuaga, J.L., Bermúdez de Castro, J.M., Carbonell, E., Evaluating the suitability of extended-range luminescence dating techniques over Early and Middle Pleistocene timescales: Published datasets and case studies from Atapuerca, Spain. Quaternary International, submitted.

Demuro, M., Arnold, L.J., Froese, D.G., Roberts, R.G., 2013. OSL dating of loess deposits bracketing Sheep Creek tephra beds, northwest Canada: Dim and problematic single-grain OSL characteristics and their effect on multi-grain age estimates. Quaternary Geochronology 15, 67–87.

Duller, G.A.T., 2003. Distinguishing quartz and feldspar in single grain luminescence measurements. Radiation Measurements 37, 161–165.

| Samples | Unit | Archaeo-palaeontological layer | Description |
| --- | --- | --- | --- |
| AT10-2 | GIV | TG12 | Homogeneous and massive red silts and clays devoid of boulders but containing mudballs. |
| ATG10-1 | GIII | TG11 (GSU2) | Fairly homogeneous red sandy silt and clay layer intercalated with “paella” to the right and overlain by thicker cemented “paella” deposits. |
| ATG10-3 | GIII | TG11 (GSU7) | Fairly homogenous sandy silts and clays intercalated with <5 cm-thick beds of “paella”. Similar to ATG10-1. |
| ATG10-7 | GIII | TG10A | Homogenous, blocky red sandy silts and clays with laminar bedding features. |
| ATG10-8 | GII | TN2A | Collected from within a 7-cm thick, black and white guano deposit. Directly underlain by homogeneous red clays. |
| ATG10-9 | GII | TG7 | 45 cm-thick yellowish light brown sandy silts and clays. |
| ATG10-10 | GII | TG7 | 30 cm-thick yellowish light brown sandy silts and clays without boulders. |
| ATZ10-4 | GII/GI | -- | 120 cm-thick, orange-brown, homogeneous, bedded sandy silts and clays. |
| ATZ10-3 | GI | -- | 45 cm-thick, dark brown, very homogeneous sandy silts and clays. |
| ATG10-4 | GI | -- | Well-bedded, 50 cm-thick unit of homogeneous light brown sandy silts and clays. Unit shows dipping from left to right. |

Table S1. Details of the Galería luminescence dating samples analysed in this study.

**U-238 series Th-232 series**

**Sample** U-238 (Bq kg^-1^) Ra-226 (Bq kg^-1^) Pb-210 (Bq kg^-1^) **Ra-226:U-238 Pb-210:Ra-226** Ra-228 (Bq kg^-1^) Th-228 (Bq kg^-1^) **Th-228:Ra-228**

AT10-2 29.45±4.81 26.61±1.95 25.06±2.11 **0.90±0.16 0.94±0.11** 47.76±3.32 48.51±4.04 **0.98±0.11**

ATG10-1 34.28±5.22 29.27±2.13 27.51±2.30 **0.85±0.14 0.94±0.10** 47.10±3.26 47.01±3.91 **1.00±0.11**

ATG10-3 29.71±4.01 30.93±2.51 31.03±2.59 **1.04±0.16 1.00±0.12** 53.93±3.85 55.84±4.66 **0.97±0.11**

ATG10-7 36.80±4.83 33.66±2.72 32.73±2.71 **0.92±0.14 0.97±0.12** 63.52±4.49 63.54±5.29 **1.00±0.11**

ATG10-8 32.19±4.34 33.72±2.72 35.51±2.92 **1.05±0.17 1.05±0.12** 73.66±1.35 71.93±5.97 **1.02±0.11**

ATG10-9 31.58±4.24 32.23±2.61 32.79±2.70 **1.02±0.16 1.02±0.12** 62.19±4.38 63.90±5.31 **0.97±0.11**

ATG10-10 36.79±5.29 30.87±2.25 31.87±2.66 **0.84±0.14 1.03±0.11** 53.65±3.71 53.82±4.48 **0.99±0.11**

ATZ10-4 37.32±5.65 32.06±2.35 32.92±2.75 **0.86±0.14 1.03±0.11** 60.96±4.25 64.20±5.38 **0.95±0.10**

ATZ10-3 40.81±5.45 37.94±3.06 39.49±3.24 **0.93±0.15 1.04±0.12** 68.19±4.81 70.45±5.85 **0.97±0.11**

ATG10-4 39.39±6.02 38.40±2.74 38.77±320 **0.98±0.16 1.01±0.11** 58.50±4.02 59.47±4.93 **0.98±0.11**

Table S2. Radionuclide activities (Bq kg^-1^) and daughter-to-parent ratios obtained from high resolution gamma spectrometry (HRGS) measurements of the ^238^U and ^232^Th decay chains.

| Stratigraphic Unit | GIV | GIIIb | GIIIb | GIIIa | GIIa | GIIa | GIIa | TZ | GIb |  |
| --- | --- | --- | --- | --- | --- | --- | --- | --- | --- | --- |
| Archaeo-paleontological Level | TG12 | TG11 | TG11 | TG10-A | TNB2 | TG7 | TG7 |  |  |  |
| **Sample name** | **AT10-2** | **ATG10-1** | **ATG10-3** | **ATG10-7** | **ATG10-8** | **ATG10-9** | **ATG10-10** | **ATZ10-4** | **ATG10-4** |  |
| Total number of grain-hole positions measured | 1000 | 800 | 800 | 1100 | 1200 | 600 | 700 | 600 | 1700 |  |
|  | % | % | % | % | % | % | % | % | % |  |
| T_n_ <3σ background (non-luminescent grains) | 75.4 | 67.5 | 65.1 | 58.5 | 69.6 | 83.2 | 84.0 | 77.7 | 86.5 |  |
| Poor recycling ratio | 3.0 | 4.8 | 5.0 | 6.4 | 5.7 | 3.2 | 2.6 | 2.7 | 3.1 |  |
| Poor OSL IR depletion ratio | 0 | 0.1 | 0 | 0 | 0.1 | 0 | 0.4 | 0.2 | 0 |  |
| Recuperation (0 Gy L_i_/T_i_ >5% of L_n_/T_n_) | 0.6 | 0.5 | 0.6 | 0.7 | 0.6 | 0.2 | 0.1 | 0 | 0.1 |  |
| Relative error on the net T_n_ signal >30% | 16.7 | 19.9 | 22.1 | 21.7 | 16.8 | 9.3 | 10.1 | 15.3 | 8.9 |  |
| Anomalous dose-response / unable to perform Monte Carlo fit ^a^ | 0.7 | 1.3 | 0.8 | 2.3 | 0.8 | 0.7 | 0.4 | 0.3 | <0.1 |  |
| Non-intercepting grains (L_n_/T_n_ > dose-response curve saturation) | 0.3 | 0.1 | 0.6 | 1.4 | 0.4 | 0.5 | 0.3 | 0.2 | 0.2 |  |
| Relative error of D_e_ >50% | 0 | 0 | 0 | 0 | <0.1 | 0 | 0.3 | 0.2 | 0 |  |
| Saturated grains (L_n_/T_n_ ≈ dose-response curve saturation) | 0 | 0 | 0.4 | 0.3 | 0.5 | 0.2 | 0 | 0 | 0 |  |
|  |  |  |  |  |  |  |  |  |  |  |
| **Accepted grains (used for D_e_ determination)** | **3.3** | **5.8** | **5.4** | **8.5** | **5.6** | **2.8** | **1.7** | **3.5** | **1.1** |  |

Table S3. Single-grain TT-OSL classification statistics for the Galería samples

|  |  |  |  |  |  |  |  |  |  |  |  |  |  |  |
| --- | --- | --- | --- | --- | --- | --- | --- | --- | --- | --- | --- | --- | --- | --- |
| **Sample** | **Mineral/Signal** | **Grains per disc / grain-hole** | **Grain size (µm)** | **Given dose (Gy)** | **Bleaching** | **Residual (non-dosed) assessment** | | |  | **Dose recovery test** | |  |  |  |
|  |  |  |  |  |  | **accepted/measured** | **Recycling ratio** | **W-mean D_e_ (Gy)** | **OD (%)** | **accepted/measured** | **Recycling ratio** | **W-mean D_e_ (Gy)** | **OD (%)** | **Net measured/ given dose ratio** |
| ATG10-3 | quartz / TT-OSL | ~4400 | 90-125 | 550 ± 11 | 3.5 weeks sunlight | 2 / 2 | 0.99 ± 0.01 | 182 ± 5 | 0 ± 0 | 3 / 4 | 1.05 ± 0.03 | 845 ± 32 | 3 ± 2 | 1.21 ± 0.06 |
| ATG10-3 | quartz / TT-OSL | ~4400 | 90-125 | 550 ± 11 | None | 4 / 4 | 1.05 ± 0.02 | 681 ± 36 | 10 ± 4 | 5 / 5 | 1.05 ± 0.02 | 1217 ± 125 | 23 ± 7 | 0.98 ± 0.11 |
|  |  |  |  |  |  |  |  |  |  |  |  |  |  |  |
| ATG10-3 | quartz / TT-OSL | ~18  (single-grain measurement) | 90-125 | 554 ± 12 | 6 weeks sunlight | 37 / 400 | 1.04 ± 0.03 | 92 ± 3 | 55 ± 10 | 38 / 500 | 1.05 ± 0.04 | 696 ± 26 | 9 ± 5 | 1.09 ± 0.05 |
|  |  |  |  |  |  |  |  |  |  |  |  |  |  |  |
| ATG10-3 | K-feldspar / pIR-IR_225_ | ~360 | 90-125 | 600 ± 12 | 1.5 hours in SOL2 | 2 / 2 | 1.01 ± 0.01 | 13 ± 1 | 4 ± 3 | 3 / 3 | 1.00 ± 0.01 | 614 ± 12 | 10 ± 5 | 1.00 ± 0.03 |
| ATG10-3 | K-feldspar / pIR-IR_255_ | ~360 | 90-125 | 600 ± 12 | 1.5 hours in SOL2 |  | ± |  |  |  |  |  |  |  |
| ATG10-3 | K-feldspar / pIR-IR_290_ | ~360 | 90-125 | 600 ± 12 | 1.5 hours in SOL2 | 2 / 2 | 0.99 ± 0.02 | 30 ± 1 | 0 ± 0 | 5 / 5 | 0.98 ± 0.01 | 683 ± 25 | 7 ± 3 | 1.09 ± 0.05 |
|  |  |  |  |  |  |  |  |  |  |  |  |  |  |  |
| ATG10-10 | K-feldspar / pIR-IR_225_ | ~360 | 90-125 | 1000 | 1.5 hours in SOL2 | 2 / 2 | 0.99 ± 0.01 | 15 ± 1 | 6 ± 3 | 3 / 3 | 0.99 ± 0.01 | 994 ± 20 | 0 ± 0 | 0.97 ± 0.03 |
| ATG10-10 | K-feldspar / pIR-IR_255_ | ~360 | 90-125 | 1000 | 1.5 hours in SOL2 | 2 / 2 | 1.00 ± 0.01 | 24 ± 1 | 7 ± 4 | 3 / 3 | 0.98 ± 0.01 | 1191 ± 34 | 0 ± 0 | 1.17 ± 0.04 |
| ATG10-10 | K-feldspar / pIR-IR_290_ | ~360 | 90-125 | 1000 | 1.5 hours in SOL2 | 2 / 2 | 1.02 ± 0.02 | 37 ± 2 | 8 ± 4 | 3 / 3 | 0.97 ± 0.02 | 1165 ± 57 | 6 ± 5 | 1.13 ± 0.06 |
|  |  |  |  |  |  |  |  |  |  |  |  |  |  |  |

Table S4. Results obtained for the dose recovery tests performed using protocol A of Table 4 for the TT-OSL signal.

| **Sample name** | **ATG10-3** | **ATG10-3** |
| --- | --- | --- |
|  | Sun-bleached grains  (residual) | Dosed grains |
| Total number of grain-hole positions measured | 400 | 500 |
|  | % | % |
| T_n_ <3σ background (non-luminescent grains) | 69.0 | 68.2 |
| Poor recycling ratio | 4.0 | 4.2 |
| Poor OSL IR depletion ratio | 0 | 0 |
| Recuperation (0 Gy L_i_/T_i_ >5% of L_n_/T_n_) | 1.5 | 1.0 |
| Relative error on the net T_n_ signal >30% | 16.3 | 16.6 |
| Anomalous dose-response / unable to perform Monte Carlo fit ^a^ | 0 | 1.2 |
| Non-intercepting grains (L_n_/T_n_ > dose-response curve saturation) | 0 | 0.8 |
| Relative error of D_e_ >50% | 0 | 0.4 |
| Saturated grains (L_n_/T_n_ ≈ dose-response curve saturation) | 0 | 0 |
|  |  |  |
| **Accepted grains (used for D_e_ determination)** | **9.3** | **7.6** |

Table S5. Rejection statistics from the single-grain TT-OSL dose recovery test performed on samples ATG10-3 corresponding to Table S4.

A

B

Figure S1. (A) Cumulative light-sum plots for the Galería samples constructed from the ranked net natural test dose signal (T_n_) (using the first 0.24 s of laser stimulation minus a background-subtraction from the final 0.25 s). Plot (B) shows ranked signal counts normalised to the given T_n_ dose (200-300 Gy). Data shown is for single-grain TT-OSL measurements made using the 90-125 µm grain fraction (~18 grains per hole; Arnold et al., 2012).

A

B

C

D

Figure S2. Single-grain TT-OSL dose recovery test results (~18 grains per hole). Quartz grains from sample ATG10-3 were exposed to direct sunlight for ~6 weeks. Measured residual doses (A, B) and D_e_ values measured for accepted grains after administering a laboratory dose of ~554 Gy (C, D). The grey bars on the radial plots are centred on the weighted mean D_e_ values given in **Table S4**.

Figure S3. Histogram showing the distribution of single-grain TT-OSL D_0_ values for the Galería samples. The D_0_ values shown here equate to the dose value for which the dose-response curve slope is 1/*e* (or ~0.37) of its initial value. D_0_ values are shown for grains that could be fitted with a single-saturating exponential dose-response curve (*n* = 270). An additional 80 grains (not included in this dataset) displayed linear dose-response curves with indeterminately high dose saturation limits.

Figure S4. Single-grain TT-OSL D_e_ distributions for the Galería samples, shown as histograms (left column) and radial plots (right column). The D_e_ datasets of the four samples not included here (AT10-2, ATG10-7, ATG10-3 and ATZ10-4) are shown in **Figure 5** of the main text.

I

J

A

B

C

D

G

H

E

F

ATG10-1

ATG10-8

ATG10-4

ATG10-10

ATG10-9

ATG10-9

CAM: 930 ± 19 Gy

OD: 4 ± 2%

ATG10-1

CAM: 658 ± 7 Gy

OD: 0 ± 0%

A

C

ATZ10-3

CAM: 1131 ± 72 Gy

OD: 14 ± 5%

ATZ10-4

CAM: 1054 ± 15 Gy

OD: 0 ± 0%

ATG10-10

CAM: 996 ± 27 Gy

OD: 6 ± 2%

D

E

F

ATG10-3

CAM: 629 ± 8 Gy

OD: 2 ± 2%

B

AT10-2

CAM: 679 ± 23 Gy

OD: 7 ± 3%

G

Figure S5. Radial plots showing the pIR-IR_225_ D_e_ distributions of the remaining Galería samples not included in **Figure 6** of the main text.
